# Interactions Between Monocarboxylate Transporter *MCT1* Gene Variants and the Kinetics of Blood Lactate Production and Removal After High-Intensity Efforts: A Cross-Sectional Study

**DOI:** 10.3390/genes16101160

**Published:** 2025-09-30

**Authors:** Ewelina Maculewicz, Andrzej Mastalerz, Anna Mróz, Monika Johne, Katarzyna Krawczak-Wójcik, Agata Pabin, Aleksandra Garbacz, Katarzyna Komar, Myosotis Massidda, Petr Stastny, Aleksandra Bojarczuk

**Affiliations:** 1 Faculty of Physical Education, Jozef Pilsudski University of Physical Education in Warsaw, 00-968 Warsaw, Poland; ewelina.maculewicz@awf.edu.pl (E.M.); andrzej.mastalerz@awf.edu.pl (A.M.); anna.mroz@awf.edu.pl (A.M.); monika.johne@awf.edu.pl (M.J.); katarzyna.krawczak@awf.edu.pl (K.K.-W.); 2 Department of Laboratory Diagnostics, Military Institute of Aviation Medicine, 01-755 Warsaw, Poland; apabin@wiml.waw.pl (A.P.); kkomar@wiml.waw.pl (K.K.); 3 Faculty of Animal Genetics and Conservation, Warsaw University of Life Sciences, 02-787 Warsaw, Poland; aleksandra_garbacz@sggw.edu.pl; 4 Department of Medical Sciences and Public Health, University of Cagliari, 72-09124 Cagliari, Italy; myosotis.massidda@unica.it; 5 Faculty of Physical Education and Sport, Charles University, 162-52 Prague, Czech Republic; stastny@ftvs.cuni.cz; 6 Faculty of Physical Education, Gdansk University of Physical Education and Sport, 80-336 Gdansk, Poland

**Keywords:** MCT1, genotype, lactate kinetics, polymorphisms, Wingate

## Abstract

Background/Objectives: Lactate (LA) is a key metabolite in exercise metabolism, transported across cell membranes by monocarboxylate transporters (MCTs). Although genetic variation in *MCT* genes has been linked to LA kinetics, evidence in athletic populations remains limited. This study investigated nine MCT1 polymorphisms (rs4301628, rs12028967, rs10857983, rs3789592, rs10776763, rs1049434, rs6537765, rs7556664, rs7169) in relation to LA metabolism. Methods: 337 Polish and Czech males (elite athletes, sub-elite competitors, physically active controls) performed two maximal Wingate tests. Buccal swabs were collected for DNA extraction and single nucleotide polymorphism (SNP) genotyping. LA was assessed before and after the tests. Results: Five variants (rs3789592, rs7556664, rs7169, rs1049434, rs6537765) remained significantly associated with LA measured 30 min after the second Wingate (LA30′) and delta clearance capacity (DCC) in elites (codominant and recessive models: *p* = 0.01–0.03; false discovery rate (FDR)-adjusted *p* = 0.02–0.04). Rs10776763 showed the broadest associations, surviving FDR for LA30′ in all models (*p* = 0.003–0.03; FDR-adjusted *p* = 0.01–0.03) and for LA accumulation capacity (ACC) in the recessive model (*p* = 0.01; FDR-adjusted *p* = 0.03). Rs12028967 also supported a clearance role, with LA30′ significant in elites (*p* = 0.004; FDR-adjusted *p* = 0.01) and DCC in the overall cohort (*p* = 0.02; FDR-adjusted *p* = 0.03). In contrast, rs4301628 and rs10857983 demonstrated isolated LA30′ effects in elites (*p* = 0.004–0.01; FDR-adjusted *p* = 0.01), and no production-phase endpoint other than rs10776763 survived FDR; ACC remained significant in the recessive model (*p* = 0.01; FDR-adjusted *p* = 0.03). Conclusions: The results suggest that *MCT1* polymorphisms contribute to differences in LA metabolism and warrant replication in larger, more diverse cohorts.

## 1. Introduction

The efficiency of blood lactate (LA) accumulation and clearance is a critical determinant of performance and recovery in anaerobic sports [1,2,3,4,5,6]. Present exercise training methodologies utilize LA concentrations to evaluate physiological demands, offering valuable information on the ideal exercise intensities suited for various populations [7,8]. Although acute LA responses to effort are well characterized physiologically [9], interindividual differences remain significant and may reflect underlying genetic factors, particularly in proteins that mediate LA transport [2,10,11,12].

Monocarboxylate transporter 1 (MCT1), encoded by the *SLC16A1* gene, is responsible for shuttling LA and hydrogen ions across cell membranes, thereby influencing intracellular pH and LA accumulation [13,14]. MCT1 is expressed in all tissues, with the exception of pancreatic endocrine β cells [15]. *MCT1* gene polymorphisms have been linked to performance traits [2,10,11,12,16], but few have been associated with LA metabolism in athletes [2,17,18,19,20,21]. However, the majority of research to date has focused on single *MCT1* polymorphisms in the context of LA utilization, often in isolation, without considering broader patterns across multiple variants or comparing associations between different athletic performance levels [12,17,19,20,21,22,23,24]. Given the physiological importance of LA kinetics for exercise performance and recovery, it is valuable to explore how specific *MCT1* genotypes contribute to individual variation in LA accumulation and removal. These insights could help explain individual differences in fatigue tolerance and recovery, allowing for more personalized approaches to training. This study investigated nine single nucleotide polymorphisms (SNPs) in the *MCT1* gene (rs4301628, rs12028967, rs10857983, rs3789592, rs7556664, rs7169, rs1049434, rs10776763, and rs6537765) and their associations with LA kinetics following repeated all-out anaerobic efforts. Currently, no citation records are available in the National Center for Biotechnology Information (NCBI) dbSNP database for the following six variants: rs4301628, rs12028967, rs10857983, rs3789592, rs10776763, and rs6537765 [25]. In contrast, rs7556664 and rs7169 have been linked with improved survival in multiple myeloma patients [26], though not in exercise-related contexts. Rs1049434 has been frequently investigated in relation to sport-related traits and LA metabolism [11,17,18,19,20,22,27,28,29]. Notably, all nine variants were previously analyzed in our recent exploratory study involving elite male athletes, where selected SNPs were implicated in gene–gene interactions and haplotypes affecting LA accumulation and clearance following two intermittent all-out anaerobic Wingate tests [2]. That study identified several promising genotype combinations and haplotype patterns, particularly involving *MCT1*, *MCT2*, and *MCT4*, which were associated with altered LA accumulation and clearance, despite limited functional annotations in public databases [2]. However, because the analysis emphasized interactive and haplotypic effects, the independent contributions of each *MCT1* variant and their haplotypes to LA responses remained to be elucidated. In the present study, we revisited these same nine SNPs with a targeted focus on their independent and combined effects within the *MCT1* gene alone.

This study aimed to investigate the interactions between specific variants of the *MCT1* gene and the kinetics of blood LA production and clearance following high-intensity efforts. Using a two-bout Wingate protocol, we evaluated LA production, accumulation capacity, and clearance in male individuals across elite and sub-elite sprinters and physically active (control) groups. We hypothesized that specific *MCT1* genotypes are associated with distinct patterns in LA accumulation and removal, which may vary based on an athlete’s training level. We examined these associations to enhance understanding of genetic influences on LA metabolism in elite and sub-elite sprinters.

## 2. Materials and Methods

### 2.1. Study Design

**Figure 1:**
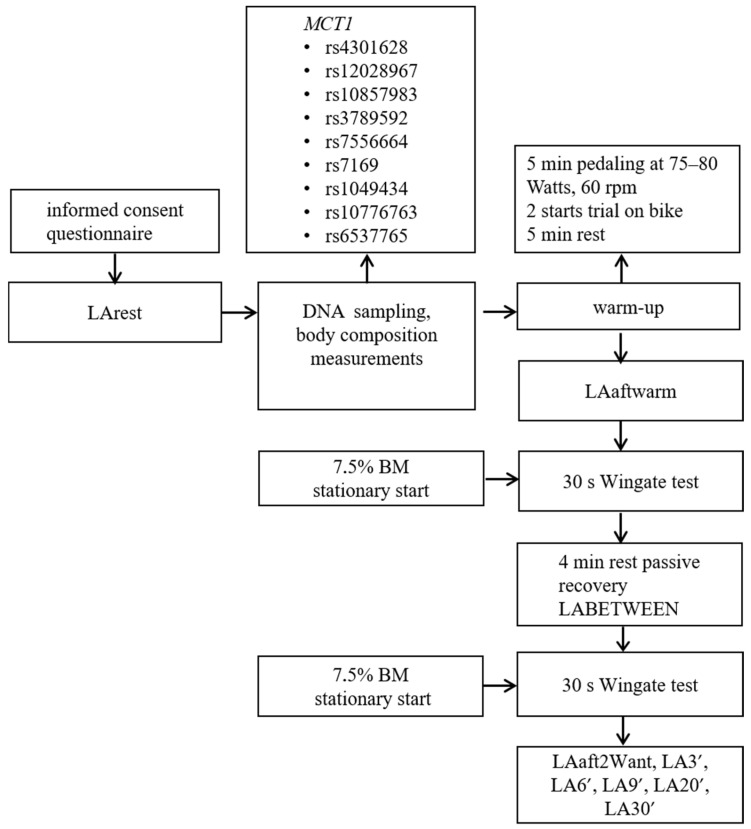
Flow chart of formal procedures, DNA sampling, anaerobic loading, and timing of blood LA samples. LArest—LA at rest, BM—body mass, LAaftwarm—LA following the warm-up, LABETWEEN-LA during the third minute of rest after the first Wingate test but before the second, LAaft2Want—LA immediately after the second Wingate; LA3’—LA at 3’ min post-second Wingate; LA6’—LA at 6’ min post-second Wingate, LA9’—LA at 9’ min post-second Wingate, LA20’—LA at 20’ min post-second Wingate; LA30’—LA at 30’ min after the second Wingate test; MAX LA—maximum LA concentration.

The study design and exercise protocol followed the same procedures as previously described in detail by Maculewicz et al. (2025) [30]. In brief, the study employed a cross-sectional case–control design to explore how individual genetic profiles relate to immediate responses to intense glycolytic challenge, with specific focus on LA dynamics during and after exercise. Participants performed two intermittent all-out Wingate tests, with LA concentrations measured at predefined time points and DNA collected before exercise (Figure 1). The two maximal intermittent anaerobic bouts were carried out in the Physiological Laboratory of the University of Physical Education in Warsaw and the Biomedical Laboratory at the Faculty of Physical Education and Sport at Charles University, as described previously [30].

### 2.2. Participants

The participant cohort, inclusion and exclusion criteria, and descriptive characteristics were identical to those reported in our recent publication [30]. Briefly, the study included 337 male athletes (42 elite, 103 sub-elite, and 192 physically active controls) aged 16–29 years from Poland and the Czech Republic. All procedures regarding eligibility, training status, and health verification followed the same standardized methodology as described previously [30].

### 2.3. Intermittent All-Out Wingate Tests

The Wingate test protocol, including warm-up, rest, and recovery procedures, was identical to that outlined in our earlier work [30]. In short, participants performed a standardized 5-min (min) warm-up on a cycling ergometer (Monark 894 E peak bike, Varberg, Sweden), followed by two successive 30-s all-out Wingate tests with resistance set at 7.5% of body mass. Passive recovery (4 min) was introduced between bouts, and an active recovery phase (4 min at ~50 rpm) followed the second test [30].

### 2.4. Blood LA Measurement

Capillary blood sampling and LA measurements were performed according to the standardized protocol previously reported by Maculewicz et al. (2025) [30]. In brief, LA was measured at nine predefined time points from fingertip capillary blood using the Biosen C-Line analyzer (EKF Diagnostics, Barleben, Germany) (Figure 1). Regarding LA measurements, identical procedures were applied in both laboratories (Warsaw and Prague), i.e., capillary blood was collected from the fingertip at predefined time points, and LA concentrations were determined using the same LA analyzer, with regular calibration according to the manufacturer’s guidelines. This ensured methodological consistency across sites.

### 2.5. DNA Sampling and Isolation

Buccal cell collection and DNA extraction procedures followed the same protocols as those reported in our earlier work [30].

### 2.6. Genotyping Analyses

Genotyping procedures followed the same Real-Time PCR protocol as outline in [30]. In the present study, we analyzed nine SNPs of the *MCT1* gene: seven using commercial TaqMan™ probes: rs4301628 (C_27970752_10), rs12028967 (C_26628781_10), rs10857983 (C_26628778_10), rs3789592 (C_26628769_10), and rs7556664 (C_26628970_20), rs1049434 (C_2017662_30), rs7169 (C_2017661_30) (Applied Biosystems, Waltham, MA, USA), which include starters and fluorescently labeled (VIC and FAM) MGBTM allele detection probes. The other two assays were custom, non-commercial primer and probe sets targeting the rs10776763 and rs6537765 SNPs, designed using the Custom TaqMan Assay Design Tool (Thermo Fisher Scientific, Waltham, MA, USA). Genotyping analyses were performed on anonymized samples, and the laboratory staff were blinded to the participants’ group allocation (elite, sub-elite, or physically active).

### 2.7. Statistical Analyses

Statistical procedures largely followed those applied in our previous study [30]. Normally distributed data were analyzed using one-way ANOVA to assess the influences of genotypes (rs4301628, rs12028967, rs10857983, rs3789592, rs7556664, rs7169, rs1049434, rs10776763, rs6537765) on performance-related LA parameters, including peak post-exercise LA (MAX LA), LA accumulation capacity (ACC), delta clearance capacity (DCC), and final LA concentration (LA30’). Post-hoc comparisons were performed using Fisher’s Least Significant Difference (LSD) test. For a two-group comparison, *t*-tests were used.

Genetic association was analyzed under three inheritance models: codominant (A/A vs. A/a vs. a/a), dominant (A/A vs. (A/a + a/a)), and recessive ((A/A + A/a) vs. a/a). The additive model, although often regarded as parsimonious and biologically plausible, was not applied because the cumulative effect of alleles was not the primary focus of this study. Instead, genotype-specific effects were examined. This strategy was chosen to reduce the risk of model misspecification, to capture a broader spectrum of possible genotype–phenotype relationships, and to ensure methodological consistency with our recent analysis of *MCT2* and *MCT4* polymorphisms [30]. Hardy–Weinberg equilibrium (HWE) was assessed using R software (version 4.4.3, https://cran.r-project.org). Genotype × training level interactions were not statistically tested. Statistical significance was set at α < 0.05. In addition, effect sizes (η^2^, d) with corresponding confidence intervals (CIs) were calculated, and post hoc statistical power was assessed. To account for multiple testing, a false discovery rate (FDR) adjustment was applied. All analyses were conducted using R software (version 4.4.3, https://cran.r-project.org).

## 3. Results

Supplementary Table S1 reports the data for LA concentrations measured at multiple time points across the examined genotypes and groups. The time points assessed include: baseline (LArest), post-warm-up (LAaftwarm), after the first Wingate test but before the second (LABETWEEN), immediately following the second Wingate (LAaft2WAnt), and during the recovery phase at 3, 6, 9, 20, and 30 min (LA3′, LA6′, LA9′, LA20′, LA30′).

### 3.1. Minor Allele Frequencies and Hardy–Weinberg Equilibrium

The minor allele frequencies (MAFs) for the analyzed SNPs ranged from 33.09% (rs12028967) to 37.69% (rs6537765), indicating that all loci were polymorphic and suitable for association analysis in this cohort (Table 1). All marker variants were tested for Hardy–Weinberg equilibrium (HWE). In the overall group, including all observations, the *p*-values ranged from 0.70 to 1.00, confirming that all loci were in HWE. In the elite group, the *p*-values ranged from 0.73 to 1.00. Within the sub-elite group, all variants showed *p*-values between 0.05 and 0.31, which also supports the HWE assumptions. In the physically active group, *p*-values ranged from 0.05 to 0.31, confirming adherence to HWE. In the overall group, *p*-values ranged from 0.70 to 1.00. Although the rs10776763 variant showed a borderline *p*-value of 0.05 in the sub-elite and physically active subgroups, no significant deviation from HWE was confirmed. Therefore, all analyzed SNPs were retained for further analyses (Table 1).

### 3.2. Maximal LA Accumulation

**Figure 2:**
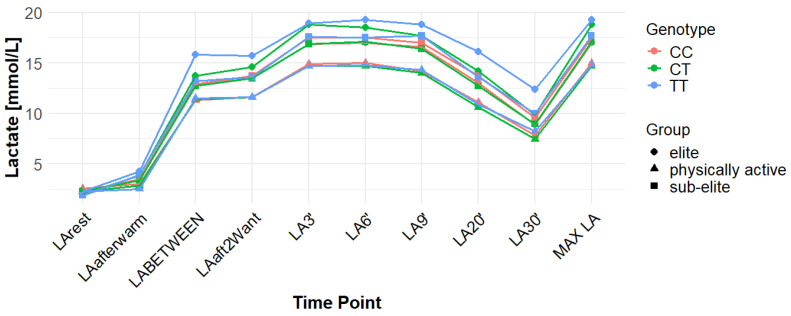
Time course of lactate (LA) concentrations across genotypes for the *MCT1* (monocarboxylate transporter 1) rs4301628 polymorphism. The data are presented as the mean for each time point. LA—lactate; LArest—LA concentration before the warm-up at rest; LAaftwarm—LA after warm-up; LABETWEEN—LA in the third minute of rest after the first but before the second Wingate test; LAaft2Want—LA immediately after the second Wingate test; LA3’—LA at 3’ min post-second Wingate; LA6’—LA at 6’ min post-second Wingate, LA9’—LA at 9’ min post-second Wingate, LA20’—LA at 20’ min post-second Wingate; LA30’—LA at 30’ min after the second Wingate test; MAX LA—maximum LA concentration; CC—homozygote for the major allele (C); CT—heterozygote; TT—homozygote for the minor allele (T).

**Figure 3:**
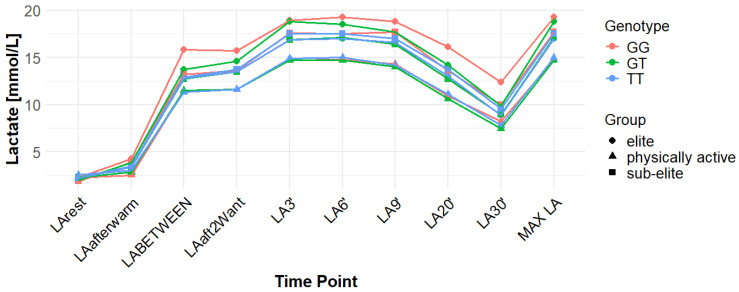
Time course of lactate (LA) concentrations across genotypes for the *MCT1* (monocarboxylate transporter 1) rs12028967 polymorphism. The data are presented as the mean for each time point. LA—lactate; LArest—LA concentration before the warm-up at rest; LAaftwarm—LA after warm-up; LABETWEEN—LA in the third minute of rest after the first but before the second Wingate test; LAaft2Want—LA immediately after the second Wingate test; LA3’—LA at 3’ min post-second Wingate; LA6’—LA at 6’ min post-second Wingate, LA9’—LA at 9’ min post-second Wingate, LA20’—LA at 20’ min post-second Wingate; LA30’—LA at 30’ min after the second Wingate test; MAX LA—maximum LA concentration; GG—homozygote for the minor allele (G); GT—heterozygote; TT—homozygote for the major allele (T).

**Figure 4:**
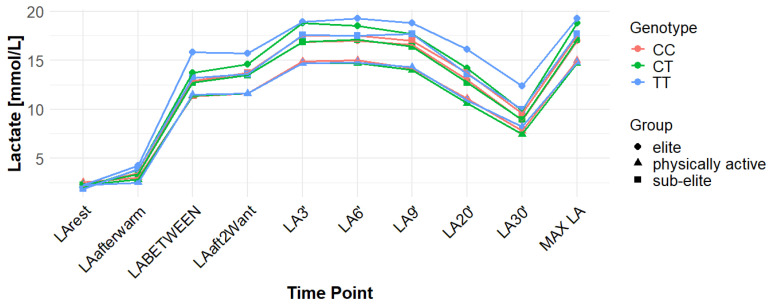
Time course of lactate (LA) concentrations across genotypes for the *MCT1* (monocarboxylate transporter 1) rs10857983 polymorphism. The data are presented as the mean for each time point. LA—lactate; LArest—LA concentration before the warm-up at rest; LAaftwarm—LA after warm-up; LABETWEEN—LA in the third minute of rest after the first but before the second Wingate test; LAaft2Want—LA immediately after the second Wingate test; LA3’—LA at 3’ min post-second Wingate; LA6’—LA at 6’ min post-second Wingate, LA9’—LA at 9’ min post-second Wingate, LA20’—LA at 20’ min post-second Wingate; LA30’—LA at 30’ min after the second Wingate test; MAX LA—maximum LA concentration; CC—homozygote for the major allele (C); CT—heterozygote; TT—homozygote for the minor allele (T).

**Figure 5:**
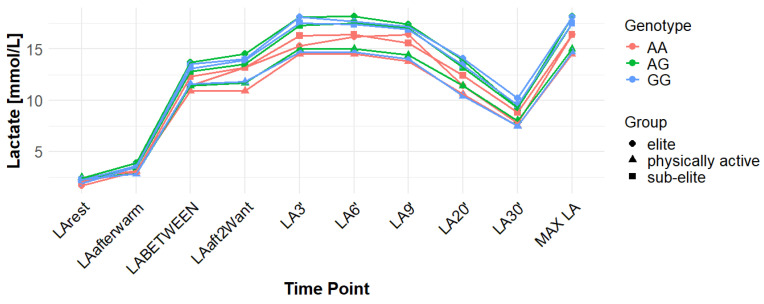
Time course of lactate (LA) concentrations across genotypes for *MCT1* (monocarboxylate transporter 1) rs3789592 polymorphism. The data are presented as the mean for each time point. LArest—LA concentration before the warm-up at rest; LAaftwarm—LA after warm-up; LABETWEEN—LA in the third minute of rest after the first but before the second Wingate test; LAaft2Want—LA immediately after the second Wingate test; LA3’—LA at 3 min post-second Wingate; LA6’—LA at 6 min post-second Wingate. LA9’—LA at 9 min post-second Wingate. LA20’—LA at 20 min post-second Wingate; LA30’—LA at 30 min after the second Wingate test; MAX LA—maximum LA concentration; AA—homozygote for the minor allele (A); AG—heterozygote; GG—homozygote for the major allele (G).

**Figure 6:**
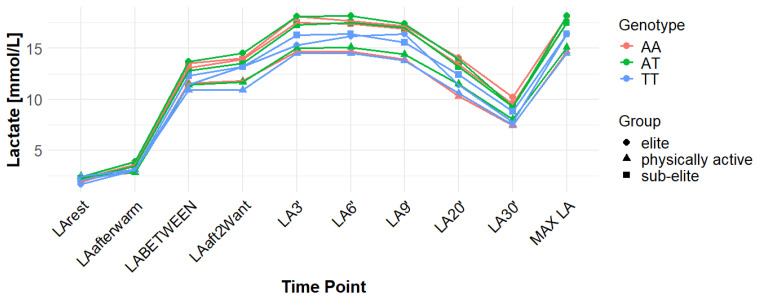
Time course of lactate (LA) concentrations across genotypes for *MCT1* (monocarboxylate transporter 1) rs7556664 polymorphism. The data are presented as the mean for each time point. LArest—LA concentration before the warm-up at rest; LAaftwarm—LA after warm-up; LABETWEEN—LA in the third minute of rest after the first but before the second Wingate test; LAaft2Want—LA immediately after the second Wingate test; LA3’—LA at 3 min post-second Wingate; LA6’—LA at 6 min post-second Wingate. LA9’—LA at 9 min post-second Wingate. LA20’—LA at 20 min post-second Wingate; LA30’—LA at 30 min after the second Wingate test; MAX LA—maximum LA concentration; AA—homozygote for the major allele (A); AT—heterozygote; TT—homozygote for the minor allele (T).

**Figure 7:**
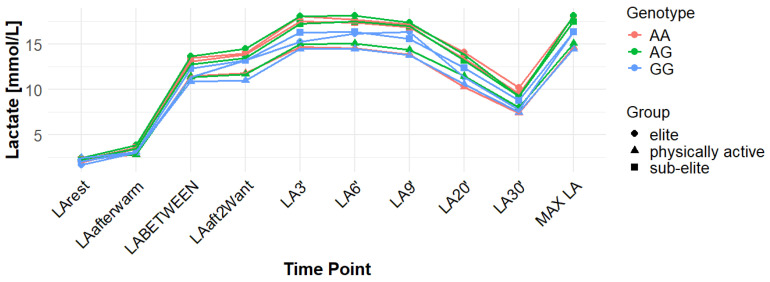
Time course of lactate (LA) concentrations across genotypes for *MCT1* (monocarboxylate transporter 1) rs7169 polymorphism. The data are presented as the mean for each time point. LArest—LA concentration before the warm-up at rest; LAaftwarm—LA after warm-up; LABETWEEN—LA in the third minute of rest after the first but before the second Wingate test; LAaft2Want—LA immediately after the second Wingate test; LA3’—LA at 3 min post-second Wingate; LA6’—LA at 6 min post-second Wingate. LA9’—LA at 9 min post-second Wingate. LA20’—LA at 20 min post-second Wingate; LA30’—LA at 30 min after the second Wingate test; MAX LA—maximum LA concentration; AA—homozygote for the major allele (A); AG—heterozygote; GG—homozygote for the minor allele (G).

**Figure 8:**
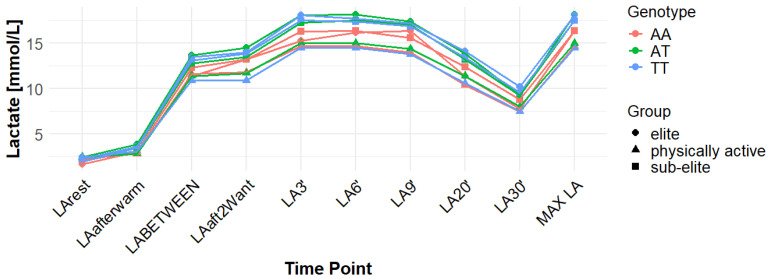
Time course of lactate (LA) concentrations across genotypes for *MCT1* (monocarboxylate transporter 1) rs1049434 polymorphism. The data are presented as the mean for each time point. LArest—LA concentration before the warm-up at rest; LAaftwarm—LA after warm-up; LABETWEEN—LA in the third minute of rest after the first but before the second Wingate test; LAaft2Want—LA immediately after the second Wingate test; LA3’—LA at 3 min post-second Wingate; LA6’—LA at 6 min post-second Wingate. LA9’—LA at 9 min post-second Wingate. LA20’—LA at 20 min post-second Wingate; LA30’—LA at 30 min after the second Wingate test; MAX LA—maximum LA concentration; AA—homozygote for the minor allele (A); AT—heterozygote; TT—homozygote for the major allele (T).

**Figure 9:**
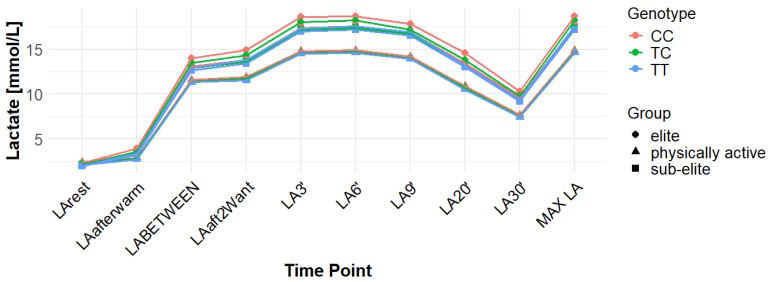
Time course of lactate (LA) concentrations across genotypes for *MCT1* (monocarboxylate transporter 1) rs10776763 polymorphism. The data are presented as the mean for each time point. LArest—LA concentration before the warm-up at rest; LAaftwarm—LA after warm-up; LABETWEEN—LA in the third minute of rest after the first but before the second Wingate test; LAaft2Want—LA immediately after the second Wingate test; LA3’—LA at 3 min post-second Wingate; LA6’—LA at 6 min post-second Wingate. LA9’—LA at 9 min post-second Wingate. LA20’—LA at 20 min post-second Wingate; LA30’—LA at 30 min after the second Wingate test; MAX LA—maximum LA concentration; CC—homozygote for the minor allele (C); TC—heterozygote; TT—homozygote for the major allele (T).

**Figure 10:**
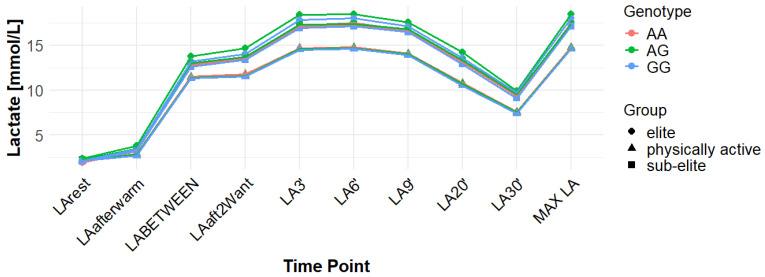
Time course of lactate (LA) concentrations across genotypes for *MCT1* (monocarboxylate transporter 1) rs6537765 polymorphism. The data are presented as the mean for each time point. LArest—LA concentration before the warm-up at rest; LAaftwarm—LA after warm-up; LABETWEEN—LA in the third minute of rest after the first but before the second Wingate test; LAaft2Want—LA immediately after the second Wingate test; LA3’—LA at 3 min post-second Wingate; LA6’—LA at 6 min post-second Wingate. LA9’—LA at 9 min post-second Wingate. LA20’—LA at 20 min post-second Wingate; LA30’—LA at 30 min after the second Wingate test; MAX LA—maximum LA concentration; AA—homozygote for the minor allele (A); AG—heterozygote; GG—homozygote for the major allele (G).

Associations with maximum LA concentration (MAX LA) were initially observed for several SNPs. However, none of these remained statistically significant after correction for false discovery rate (FDR). For *MCT1* rs10857983 associations in the overall population, were detected under the codominant (*p* = 0.04; FDR-adjusted *p* = 0.06) and recessive models (*p* = 0.03; FDR-adjusted *p* = 0.06). In the overall group, the CC genotype showed the highest mean MAX LA (16.53 mmol/L), followed by CT (16.40 mmol/L) and TT (16.14 mmol/L). Elevated LA values were also observed among TT carriers in sub-elite (17.64 mmol/L) and elite athletes (20.05 mmol/L). However, neither association for rs10857983 remained significant after FDR correction. The rs3789592 polymorphism demonstrated an association with MAX LA under the recessive model (*p* = 0.03; FDR-adjusted *p* = 0.08) in the overall group. In this group, GG (16.34 mmol/L) and GA (16.74 mmol/L) showed higher mean LA than AA (15.64 mmol/L). This association did not remain significant following FDR correction. The rs7556664 variant exhibited an association with MAX LA in the general population under the codominant (*p* = 0.04; FDR-adjusted *p* = 0.06) and recessive (*p* = 0.03; FDR-adjusted *p* = 0.06) models. AT carriers showed higher mean post-exercise LA than AA carriers in the overall cohort (16.75 vs. 16.33 mmol/L), in physically active participants (15.51 vs. 15.13 mmol/L), and in the sub-elite group (18.01 vs. 17.54 mmol/L), whereas in the elite group AT was slightly lower than AA (18.84 vs. 18.94 mmol/L). None of the associations for rs7556664 were significant after applying FDR correction. Another SNP, rs7169, was associated with MAX LA in the overall group under the recessive model (*p* = 0.03; FDR-adjusted *p* = 0.08). AA (16.33 mmol/L) and AG (16.74 mmol/L) showed higher post-exercise concentrations than GG (13.40 mmol/L). This result did not remain significant after FDR adjustment. Finally, the rs10776763 SNP showed an association with MAX LA in elite athletes under the recessive model (*p* = 0.03; FDR-adjusted *p* = 0.09). In elite athletes, TT (16.48 mmol/L) and TC (16.50 mmol/L) showed lower LA than CC (20.05 mmol/L). However, this association also did not remain significant after FDR correction. The rs4301628, rs12028967, rs1049434, and rs6537765 polymorphisms showed no significant associations with MAX LA in any of the analyzed groups or genetic models. The effect sizes and post hoc test power estimates are presented in Table 2. Figure 2, Figure 3, Figure 4, Figure 5, Figure 6, Figure 7, Figure 8, Figure 9 and Figure 10 provide a time-course visualization of LA concentrations across genotypes and training groups, including MAX LA.

### 3.3. LA Accumulation Capacity

Associations with LA accumulation capacity (the difference between MAX LA and LA at rest, LArest; ACC) were initially observed for several SNPs. However, most did not remain statistically significant after FDR correction.

For rs4301628 in elite athletes, an association was detected under a dominant model (*p* = 0.03; FDR-adjusted *p* = 0.09). CC homozygotes had a mean ACC of 15.94 mmol/L, whereas those carrying at least one T allele (CT and TT) had mean ACC values of 17.01 mmol/L and 17.67 mmol/L, respectively. This association did not remain statistically significant following FDR correction. In elite athletes, rs12028967 demonstrated an association with ACC under the dominant model (*p* = 0.03; FDR-adjusted *p* = 0.09). Elite athletes with the TT genotype had a mean ACC of 15.94 mmol/L, whereas carriers of the minor G allele—GT and GG—showed higher mean ACC values of 17.01 mmol/L and 17.67 mmol/L, respectively. This result did not remain statistically significant after FDR adjustment. No significant associations were found in other groups. For the rs10857983 polymorphism, elite athletes again showed an association under the dominant model (*p* = 0.03; FDR-adjusted *p* = 0.09) with the CC genotype presenting lower mean ACC (15.94 mmol/L) compared to carriers of the T allele-CT (17.01 mmol/L) and TT (17.67 mmol/L). In the overall cohort, an additional association was observed under the recessive model (*p* = 0.04; FDR-adjusted *p* = 0.12), with CC + CT showing higher ACC than TT (14.15 and 14.16 vs. 13.97 mmol/L). Neither association remained significant after FDR correction. The rs7556664 polymorphism demonstrated an association with ACC in the overall cohort under the recessive model (*p* = 0.04; FDR-adjusted *p* = 0.12). In this model, individuals homozygous for the minor allele (TT genotype) demonstrated lower mean ACC values compared to carriers of the A allele (AA and AT genotypes). Specifically, the mean ACC was 13.38 mmol/L for TT individuals, while AA and AT genotypes showed higher mean values of 14.13 mmol/L and 14.37 mmol/L, respectively (all). This association lost its statistical significance following FDR correction. No significant associations were observed under this model in the physically active, sub-elite, or elite subgroups (*p* = 0.24, 0.12, and 0.44, respectively).

The rs7169 polymorphism was associated with ACC in the overall cohort when analyzed using the recessive model (*p* = 0.04; FDR-adjusted *p* = 0.12). According to this model, individuals homozygous for the minor allele (GG genotype) showed lower mean ACC values compared to carriers of the A allele (AA and AG genotypes). Specifically, the mean ACC in the overall group was 13.40 mmol/L for GG individuals, while AA and AG genotypes showed higher values of 14.13 mmol/L and 14.36 mmol/L, respectively. No significant associations were observed under this model in the physically active, sub-elite, or elite subgroups (*p* = 0.23, 0.12, and 0.44, respectively).

The rs10776763 polymorphism was significantly associated with ACC in the elite athlete subgroup under the recessive model (*p* = 0.01; FDR-adjusted *p* = 0.03). According to this model, TT + TC showed lower ACC than CC (15.82 and 17.08 vs. 17.67 mmol/L). In elite athletes, mean ACC values were 17.67 mmol/L for CC, 17.08 mmol/L for TC, and 15.82 mmol/L for TT genotypes. This result remained statistically significant after FDR correction. No significant associations were observed in other groups under this model (*p* = 0.69, 0.67, and 0.37 for all, physically active, and sub-elite, respectively) (Table 3). Figure 2, Figure 3, Figure 4, Figure 5, Figure 6, Figure 7, Figure 8, Figure 9 and Figure 10 illustrate the changes in LA concentrations over time, differentiated by genotype and training groups. The visualized time course includes both resting concentrations (LArest) and MAX LA, which together constitute the components used to calculate ACC.

### 3.4. Post-Exercise LA Clearance

Associations with delta clearance capacity (DCC), defined as the difference between maximal post-exercise LA concentration and LA concentration measured 30 min after the second Wingate test (LA30’), were initially observed for several SNPs. However, only some remained significant after FDR adjustment. The rs12028967 polymorphism was associated with DCC in the overall cohort under both the codominant and recessive models (*p* = 0.02 for both and FDR-adjusted *p* = 0.03 for both). In the overall group, individuals with the GT genotype exhibited the highest mean DCC value (8.14 mmol/L), whereas GG homozygotes had the lowest (7.26 mmol/L), and TT carriers had an intermediate value (8.07 mmol/L). In the elite athlete subgroup, however, the TT genotype showed the highest DCC (9.51 mmol/L), while the GG genotype again had the lowest (7.03 mmol/L). These associations in the overall cohort remained significant after FDR correction. Associations were noted for rs3789592 in elite athletes across all models (codominant, dominant, and recessive: *p* = 0.04; FDR-adjusted *p* = 0.04). In the codominant model, AA carriers showed the highest DCC (10.99 mmol/L), compared to AG (9.30 mmol/L) and GG (8.17 mmol/L). In the dominant model, GG showed lower DCC (8.17 mmol/L) than AG and AA (9.30 and 10.99 mmol/L, respectively). In the recessive model, GG and AG showed lower DCC (8.17 and 9.30 mmol/L) compared with AA (10.99 mmol/L). These associations remained statistically significant after FDR correction. In sub-elite athletes, an association was observed under the recessive model (*p* = 0.03; FDR-adjusted *p* = 0.09). GG and AG showed higher DCC (8.34 and 8.85 mmol/L) than AA (7.29 mmol/L). However, this association did not remain significant after FDR adjustment. The results for rs7556664 demonstrated similarities to those of rs3789592, indicating a comparable pattern between the two. In sub-elite athletes, the recessive model showed an association (*p* = 0.03; FDR-adjusted *p* = 0.09), with AA and AT showing higher DCC (8.34 and 8.85 mmol/L) than TT (7.29 mmol/L). However, this association did not remain statistically significant after FDR correction. In elite athletes, significant associations were detected across all models (codominant, dominant, and recessive: *p* = 0.04; FDR-adjusted *p* = 0.04). In the codominant model, mean DCC values were 8.17 mmol/L for AA, 9.30 mmol/L for AT, and 10.99 mmol/L for TT. In the dominant model, AA showed lower DCC than (AT + TT) and in the recessive model, (AA + AT) showed lower DCC than TT (8.17 and 9.30 vs. 10.99 mmol/L). All of these associations remained statistically significant after FDR correction. A similar pattern was observed for rs7169. Significant associations were observed in elite athletes across all models (codominant, dominant, and recessive: *p* = 0.04; FDR-adjusted *p* = 0.04). Mean DCC values were 8.17 mmol/L for AA, 9.30 mmol/L for AG, and 10.99 mmol/L for GG. In the codominant model, AA showed lower values (8.17 mmol/L) compared with AG (9.30 mmol/L) and GG (10.99 mmol/L). In the dominant model, AA showed lower DCC than AG and GG. In the recessive model, AA and AG showed lower DCC (8.17 and 9.30 mmol/L) than GG (10.99 mmol/L). All of these associations remained statistically significant after FDR correction. In sub-elite athletes, an association was observed under the recessive model (*p* = 0.03; FDR-adjusted *p* = 0.09), with AA and AG showing higher mean DCC (8.34 and 8.85 mmol/L) than GG (7.29 mmol/L). However, this association did not remain statistically significant after FDR correction. For rs1049434, significant associations emerged in the recessive model for the general population (*p* = 0.03; FDR-adjusted *p* = 0.09) and sub-elite (*p* = 0.03; FDR-adjusted *p* = 0.09). In both cases, TT + AT carriers showed higher DCC compared with AA (all: 7.94 and 8.15 vs. 7.74 mmol/L; sub-elite: 8.34 and 8.85 vs. 7.29 mmol/L). However, these associations did not remain statistically significant after FDR correction. In elite athletes, significant associations were detected across all models (codominant, dominant, and recessive (*p* = 0.04; FDR-adjusted *p* = 0.04 for all). Mean DCC values were 8.17 mmol/L for TT, 9.30 mmol/L for AT, and 10.99 mmol/L for AA, and all of these associations remained significant after FDR correction. The rs10776763 polymorphism showed associations under the codominant model in elite athletes (*p* = 0.03; FDR-adjusted *p* = 0.09) and the recessive model in the general population (*p* = 0.03; FDR-adjusted *p* = 0.09). However, neither association remained statistically significant after FDR correction. Lastly, the rs6537765 SNP showed an association in the recessive model for the general population (*p* = 0.03; FDR-adjusted *p* = 0.09), but this association lost statistical significance due to FDR adjustment. In elite athletes, significant associations were detected across all models (codominant, dominant, and recessive: *p* = 0.04; FDR-adjusted *p* = 0.04). In the codominant model, the AA genotype showed a higher DCC (10.99 mmol/L) than the AG (9.28 mmol/L) and GG (8.14 mmol/L) genotypes. In the dominant model, GG showed lower DCC (8.14 mmol/L) than AG (9.28 mmol/L) and AA (10.99 mmol/L). In the recessive model, AG and GG showed lower DCC (9.28 and 8.14 mmol/L) than AA (10.99 mmol/L). All of these associations in elite athletes remained statistically significant after FDR correction (Table 4). Figure 2, Figure 3, Figure 4, Figure 5, Figure 6, Figure 7, Figure 8, Figure 9 and Figure 10 illustrate the changes in LA concentrations over time, differentiated by genotype and training groups. The visualized time course includes both the MAX LA and LA30’, which together constitute the components used to calculate delta clearance capacity (DCC).

**Table 3:** Blood LA mean LA accumulation capacity (ACC) values in mmol/L of overall, physically active, elite, and sub-elite across genotypes in *MCT1* polymorphisms, analyzed under the codominant, dominant, and recessive allele models, with their effects (η^2^, d) with 95% CI and post hoc power.

*MCT1* SNP	Genotype	All	Physically Active	Sub-Elite	Elite	Model	All *p*-Value	Physically Active *p*-Value	sub-Elite *p*-Value	Elite *p*-Value	All Effect (η^2^, d); (95% CI)	Physically Active Effect (η^2^, d); (95% CI)	Sub-Elite Effect (η^2^, d); (95% CI)	Elite Effect (η^2^, d); (95% CI); Post Hoc Power
rs4301628	CT	14.16	12.82	15.25	17.01	codominant	0.16	0.91	0.67	0.08	<0.001; (0.00 0.01)	0.001; (0.00 0.01)	0.02; (0.00 0.09)	0.16; (0.00 0.35)
	CC	14.15	12.90	15.69	15.94	dominant	0.09	0.94	0.49	0.03/0.09 *	0.01; (−0.20 0.23)	0.01; (−0.27 0.30)	0.19; (−0.20 0.58)	−0.85; (−1.47 −0.21); 0.87
	TT	13.97	13.02	15.92	17.67	recessive	0.79	0.71	0.68	0.20	0.07; (−0.27 0.41)	−0.08; (−0.48 0.33)	−0.30; (−1.07 0.47)	−0.53; (−1.71 0.65)
rs12028967	GT	14.17	12.80	15.25	17.01	codominant	0.55	0.89	0.67	0.08	<0.001; (0.00 0.01)	0.001; (0.00 0.02)	0.02; (0.00 0.09)	0.16; (0.00 0.35)
	TT	14.15	12.91	15.69	15.94	dominant	0.80	0.88	0.49	0.03/0.09 *	−0.01; (−0.22 0.21)	−0.02; (−0.31 0.26)	−0.19; (−0.58 0.20)	0.85; (0.21 1.47); 0.87
	GG	13.97	13.02	15.92	17.67	recessive	0.41	0.71	0.68	0.20	−0.07; (−0.41 0.27)	0.08; (−0.33 0.48)	0.30; (−0.47 1.07)	0.53; (−0.65 1.71)
rs10857983	CT	14.16	12.82	15.25	17.01	codominant	0.09	0.91	0.67	0.08	<0.001; (0.00 0.01)	0.001; (0.00 0.01)	0.02; (0.00 0.09)	0.16; (0.00 0.35)
	CC	14.15	12.90	15.69	15.94	dominant	0.98	0.94	0.49	0.03/0.09 *	0.01; (−0.20 0.23)	0.01; (−0.27 0.30)	0.19; (−0.20 0.58)	−0.85; (−1.47 −0.21); 0.87
	TT	13.97	13.02	15.92	17.67	recessive	0.04/0.12 *	0.71	0.68	0.20	0.07; (−0.27 0.41)	−0.08; (−0.48 0.33)	−0.30; (−1.07 0.47)	−0.53; (−1.71 0.65)
rs3789592	AG	14.36	13.01	15.80	16.38	codominant	0.10	0.48	0.24	0.54	0.01; (0.00 0.09)	0.01; (0.00 0.04)	0.02; (0.00 0.09)	0.04; (0.00 0.20)
	GG	14.14	12.91	15.41	16.86	dominant	0.96	0.88	0.85	0.31	−0.004; (−0.22 0.21)	−0.02; (−0.31 0.27)	−0.03; (−0.44 0.38)	−0.19; (−0.80 0.42)
	AA	13.38	12.43	14.44	15.94	recessive	0.05	0.24	0.12	0.44	−0.32; (0.63 −0.01)	−0.24; (−0.64 0.16)	−0.39; (−0.95 0.18)	−0.65; (−1.59 0.30)
rs7556664	AT	14.37	13.04	15.80	16.38	codominant	0.09	0.44	0.24	0.54	0.01; (0.00 0.09)	0.01; (0.00 0.04)	0.12; (0.00 0.09)	0.04; (0.00 0.20)
	TT	13.38	12.43	14.44	15.94	dominant	0.98	0.97	0.85	0.31	−0.003; (−0.22 0.22)	−0.01; (−0.29 0.28)	0.03; (−0.38 0.44)	0.19; (−0.42 0.80)
	AA	14.13	12.88	15.41	16.86	recessive	0.04/0.12 *	0.24	0.12	0.44	0.32; (0.01 0.63)	0.24; (−0.16 0.64)	0.39; (−0.18 0.95)	0.65; (−0.30 1.59)
rs7169	AG	14.36	13.06	15.80	16.38	codominant	0.10	0.41	0.24	0.54	0.01; (0.00 0.09)	0.01; (0.00 0.05)	0.02; (0.00 0.09)	0.04; (0.00 0.20)
	GG	13.40	12.42	14.44	15.94	dominant	0.97	0.88	0.85	0.31	−0.01; (−0.23 0.21)	−0.02; (−0.31 0.27)	0.03; (−0.38 0.44)	0.19; (−0.42 0.80)
	AA	14.13	12.85	15.41	16.86	recessive	0.04/0.12 *	0.23	0.12	0.44	0.32; (0.006 0.62)	0.25; (−0.16 0.65)	0.39; (−0.18 0.95)	0.65; (−0.30 1.59)
rs1049434	AT	14.37	13.04	15.80	16.38	codominant	0.92	0.44	0.24	0.54	0.01; (0.00 0.09)	0.01; (0.00 0.04)	0.02; (0.00 0.09)	0.04; (0.00 0.20)
	TT	14.13	12.88	15.41	16.86	dominant	0.93	0.97	0.85	0.31	0.003; (−0.22 0.22)	0.01; (−0.28 0.29)	−0.03; (−0.44 0.38)	−0.19; (−0.80 0.42)
	AA	13.38	12.43	14.44	15.94	recessive	0.69	0.24	0.12	0.44	−0.32; (−0.63 0.01)	−0.24; (−0.64 0.16)	−0.39; (−0.95 0.18)	−0.65; (−1.59 0.30)
rs10776763	TC	14.26	12.95	15.27	17.08	codominant	0.92	0.81	0.77	0.06	0.003; (0.00 0.04)	0.001; (0.00 0.01)	0.02; (0.00 0.09)	0.20; (0.01 0.40)
	CC	13.81	12.91	15.44	17.67	dominant	0.93	0.94	0.96	0.20	0.03; (−0.19 0.24)	0.06; (−0.23 0.35)	−0.24; (−0.64 0.15)	0.99; (0.34 162)
	TT	14.09	12.80	15.78	15.82	recessive	0.69	0.67	0.37	0.01/0.03 *	−0.14; (−0.46 0.19)	0.01; (−0.38 0.40)	0.11; (−0.61 0.83)	−0.76; (−1.94 0.44); 0.76
rs6537765	AG	14.36	13.07	15.65	16.47	codominant	0.92	0.39	0.57	0.65	0.01; (0.00 0.08)	0.01; (0.00 0.05)	0.01; (0.00 0.05)	0.04; (0.00 0.20)
	GG	14.09	12.84	15.49	16.78	dominant	0.93	0.23	0.30	0.44	0.03; (−0.19 0.25)	0.03; (−0.25 0.32)	−0.07; (−0.48 0.34)	−0.09; (−0.71 0.52)
	AA	13.50	12.42	14.79	15.94	recessive	0.69	0.82	0.99	0.46	−0.27; (−0.58 0.04)	−0.25; (−0.65 0.16)	−0.23; (−0.79 0.33)	−0.65; (−1.59 0.30)

*MCT1*—monocarboxylate transporter 1, * false discovery rate (FDR) correction; CI—confidence interval.

**Table 4:** Blood LA mean DCC values in mmol/L for all, physically active, elite, and sub-elite individuals across genotypes in *MCT1* polymorphisms, analyzed under the codominant, dominant, and recessive allele models, with their effects (η^2^, d) with 95% CI and post hoc power.

*MCT1* SNP	Genotype	All	Physically Active	Sub-Elite	Elite	Model	All *p*-Value	Physically Active *p*-Value	Sub-Elite *p*-Value	Elite *p*-Value	All Effect (η^2^, d); (95% CI)	Physically Active Effect (η^2^, d); (95% CI)	Sub-Elite Effect (η^2^, d); (95% CI)	Elite Effect (η^2^, d); (95% CI); Post Hoc Power
rs4301628	CT	8.14	7.64	8.70	8.78	codominant	0.51	0.42	0.58	0.14	0.01; (0.00 0.09)	0.01; (0.00 0.05)	0.004; (0.00 0.04)	0.04; (0.00 0.18)
	CC	8.07	7.63	8.22	9.51	dominant	0.54	0.65	0.31	0.15	0.05; (−0.17 0.26)	0.07; (−0.22 0.35)	−0.07; (−0.46 0.32)	0.17; (−0.44 0.77)
	TT	7.26	7.05	8.41	7.03	recessive	0.47	0.19	0.95	0.08	0.37; (0.03 0.71)	0.27; (−0.13 0.67)	0.18; (−0.59 0.95)	0.76; (−0.44 1.94)
rs12028967	GT	8.14	7.64	8.70	8.78	codominant	0.02/0.03 *	0.42	0.58	0.14	0.01; (0.00 0.09)	0.01; (0.00 0.05)	0.004; (0.00 0.04)	0.04; (0.00 0.18)
	TT	8.07	7.64	8.22	9.51	dominant	0.35	0.63	0.31	0.15	−0.05; (−0.26 0.17)	−0.07; (−0.36 0.21)	0.07; (−0.32 0.46)	−0.17; (−0.77 0.44)
	GG	7.26	7.05	8.41	7.03	recessive	0.02/0.03 *	0.19	0.95	0.08	−0.37; (−0.71 −0.03)	−0.27; (−0.67 0.13)	−0.18; (−0.95 0.59)	−0.76; (−1.94 0.44)
rs10857983	CT	8.14	7.64	8.70	8.78	codominant	0.50	0.42	0.58	0.14	0.01; (0.00 0.09)	0.01; (0.00 0.05)	0.004; (0.00 0.04)	0.04; (0.00 0.18)
	CC	8.07	7.63	8.22	9.51	dominant	0.67	0.65	0.31	0.15	0.05; (−0.17 0.26)	0.07; (−0.22 0.35)	−0.07; (−0.46 0.32)	0.17; (−0.44 0.77)
	TT	7.26	7.05	8.41	7.03	recessive	0.37	0.19	0.95	0.08	0.07; (0.03 0.71)	0.27; (−0.13 0.67)	0.18; (−0.59 0.95)	0.76; (−0.44 1.94)
rs3789592	AG	8.16	7.47	8.85	9.30	codominant	0.51	0.74	0.06	0.04/0.04 *	0.005; (0.00 0.05)	0.003; (0.00 0.03)	0.02; (0.00 0.10)	0.05; (0.00 0.21); 0.80
	GG	7.93	7.69	8.34	8.17	dominant	0.71	0.45	0.69	0.04/0.04 *	0.06; (−0.16 0.28)	−0.11; (−0.40 0.18)	0.15; (−0.26 0.56)	0.46; (−0.16 1.07); 0.74
	AA	7.74	7.39	7.29	10.99	recessive	0.36	0.67	0.03/0.09 *	0.04/0.04 *	−0.14; (−0.45 0.17)	−0.09; (−0.48 0.31)	−0.31; (−0.88 0.25)	−0.16; (−1.78 0.12); 0.25
rs7556664	AT	8.15	7.46	8.85	9.30	codominant	0.50	0.70	0.06	0.04/0.04 *	0.004; (0.00 0.05)	0.004; (0.00 0.03)	0.02; (0.00 0.10)	0.05; (0.00 0.21); 0.80
	TT	7.74	7.39	7.29	10.99	dominant	0.67	0.41	0.69	0.04/0.04 *	−0.05; (−0.27 0.17)	0.12; (−0.17 0.41)	−0.15; (−0.56 0.26)	−0.46; (−1.07 0.16); 0.74
	AA	7.94	7.71	8.34	8.17	recessive	0.37	0.67	0.03/0.09 *	0.04/0.04 *	0.14; (−0.17 0.45)	0.09; (−0.31 0.48)	0.31; (−0.25 0.88)	−0.16; (−1.09 0.78); 0.25
rs7169	AG	8.14	7.46	8.85	9.30	codominant	0.46	0.66	0.06	0.04/0.04 *	0.004; (0.00 0.05)	0.004; (0.00 0.03)	0.02; (0.00 0.10)	0.05; (0.00 0.21), 0.80
	GG	7.73	7.36	7.29	10.99	dominant	0.61	0.37	0.69	0.04/0.04 *	−0.04; (−0.26 0.18)	0.13; (−0.16 0.42)	−0.15; (−0.56 0.26)	−0.46; (−1.07 0.16); 0.74
	AA	7.95	7.72	8.34	8.17	recessive	0.37	0.62	0.03/0.09 *	0.04/0.04 *	0.15; (−0.16 0.45)	0.10; (−0.30 0.50)	0.31; (−0.25 0.88)	−0.16; (−1.09 0.78); 0.25
rs1049434	AT	8.15	7.46	8.85	9.30	codominant	0.09	0.70	0.06	0.04/0.04 *	0.004; (0.00 0.05)	0.003; (0.00 0.03)	0.02; (0.00 0.10)	0.05; (0.00 0.21), 0.80
	TT	7.94	7.71	8.34	8.17	dominant	0.67	0.41	0.69	0.04/0.04 *	0.05; (−0.17 0.27)	−0.12; (−0.41 0.17)	0.15; (−0.26 056)	0.46; (−0.16 1.07); 0.74
	AA	7.74	7.39	7.29	10.99	recessive	0.03/0.09 *	0.67	0.03/0.09 *	0.04/0.04 *	−0.14; (−0.45 0.17)	−0.09; (−0.48 0.31)	−0.31; (−0.88 0.25)	0.16; (−0.78 1.10); 0.25
rs10776763	TC	8.10	7.68	8.49	8.77	codominant	0.09	0.11	0.91	0.03/0.09 *	0.01; (0.00 0.10)	0.02; (0.00 0.06)	0.003; (0.00 0.04)	0.04; (0.00 0.19),0.65
	CC	7.21	6,93	8.53	7.03	dominant	0.68	0.09	0.94	0.08	−0.10; (−0.32 0.11)	−0.08; (−0.37 0.21)	−0.11; (−0.50 0.29)	−0.18; (−0.78 0.43)
	TT	8.15	7.65	8.42	9.56	recessive	0.03/0.09 *	0.58	0.86	0.13	−0.40; (−0.73 −0.08)	−0.34; (−0.73 0.05)	−0.11; (−0.84 0.61)	−0.76; (−1.94 0.44)
rs6537765	AG	8.16	7.48	8.83	9.28	codominant	0.09	0.73	0.14	0.04/0.04 *	0.004; (0.00 0.05)	0.003; (0.00 0.03)	0.02; (0.00 0.09)	0.05; (0.00 0.21), 0.80
	GG	7.90	7.69	8.23	8.14	dominant	0.67	0.62	0.12	0.04/0.04 *	0.08; (−0.14 0.30)	−0.11; (−0.40 0.18)	0.22; (−0.19 0.64)	0.47; (−0.16 1.08); 0.74
	AA	7.82	7.36	7.61	10.99	recessive	0.03/0.09 *	0.45	0.45	0.04/0.04 *	−0.10; (−0.41 0.21)	−0.10; (−0.50 0.30)	−0.14; (−0.71 0.42)	0.16; (−0.78 1.09); 0.25

*MCT1*—monocarboxylate transporter 1, * false discovery rate (FDR) correction; CI—confidence interval.

### 3.5. Final LA Concentration

The analysis of mean LA concentration measured 30 min after the second Wingate test (LA30’) revealed several significant genotype-dependent effects associated with various *MCT1* gene polymorphisms. For the rs4301628 variant, CC homozygotes in the elite athlete subgroup showed the lowest LA concentrations (8.73 mmol/L), CT individuals presented intermediate values (10.35 mmol/L), and TT homozygotes had the highest concentrations (13.02 mmol/L). Significant associations were observed in the codominant (*p* = 0.004; FDR-adjusted *p* = 0.01), dominant (*p* = 0.01; FDR-adjusted *p* = 0.01), and recessive (*p* = 0.01; FDR-adjusted *p* = 0.01) models within the elite group, and all remained statistically significant after FDR correction. Similar genotype-dependent patterns were observed for rs12028967, which demonstrated significant effects in elite athletes under codominant (*p* = 0.004; FDR-adjusted *p* = 0.01), dominant (*p* = 0.01; FDR-adjusted *p* = 0.01), and recessive (*p* = 0.01; FDR-adjusted *p* = 0.01) models. TT carriers showed the lowest LA concentrations (8.73 mmol/L), GT heterozygotes had intermediate values (10.35 mmol/L), and GG homozygotes the highest (13.02 mmol/L). All of these associations remained statistically significant after FDR correction. The rs10857983 polymorphism revealed a nearly identical distribution of results to those observed for rs4301628 and rs12028967, with CC homozygotes showing the lowest LA30′ concentrations (8.73 mmol/L), CT heterozygotes intermediate values (10.35 mmol/L), and TT homozygotes the highest (13.02 mmol/L). The codominant (*p* = 0.004; FDR-adjusted *p* = 0.01), dominant (*p* = 0.01; FDR-adjusted *p* = 0.01), and recessive (*p* = 0.01; FDR-adjusted *p* = 0.01) models all reached statistical significance. All of these associations remained statistically significant after FDR adjustment. In the codominant model (elite), mean LA30′ values were 8.73 mmol/L for CC, 10.35 mmol/L for CT, and 13.02 mmol/L for TT. In the case of rs3789592, GG homozygotes exhibited the highest LA concentrations in elite athletes (10.77 mmol/L), GA carriers showed intermediate levels (9.54 mmol/L), and AA homozygotes the lowest values (6.96 mmol/L). In the codominant model, the GG genotype showed the highest LA (10.77 mmol/L), followed by the AG genotype (9.54 mmol/L) and the AA genotype (6.96 mmol/L). Significant associations were detected in all tested models within the elite group, with the codominant model yielding a *p*-value of 0.01 (FDR-adjusted *p* = 0.02), the dominant model a *p*-value of 0.03 (FDR-adjusted *p* = 0.03), and the recessive model a *p*-value of 0.01 (FDR-adjusted *p* = 0.02). In the recessive model, (GG + GA) showed higher LA than AA (10.77 and 9.54 vs. 6.96 mmol/L). All of these associations remained statistically significant after FDR correction. The rs7556664 variant demonstrated the same genotype-dependent pattern as rs3789592, with AA homozygotes showing the highest LA30′ concentrations (10.77 mmol/L), AT carriers intermediate values (9.54 mmol/L), and TT homozygotes the lowest (6.96 mmol/L). All genetic models produced significant results in this group: codominant (*p* = 0.01; FDR-adjusted *p* = 0.02), dominant (*p* = 0.03; FDR-adjusted *p* = 0.03), and recessive (*p* = 0.01; FDR-adjusted *p* = 0.02), and all remained statistically significant after FDR correction. In the codominant model, mean LA30′ values were 10.77 mmol/L for AA, 9.54 mmol/L for AT, and 6.96 mmol/L for TT. In the dominant model, AA showed higher LA compared with (AT + TT) carriers and in the recessive model, (AA + AT) showed higher LA than TT (10.77 and 9.54 vs. 6.96 mmol/L). Comparable patterns were observed for rs7169, where AA carriers exhibited the highest LA concentrations in elite athletes (10.77 mmol/L), AG heterozygotes presented intermediate values (9.54 mmol/L), and GG homozygotes the lowest (6.96 mmol/L). All models were significant: codominant *p* = 0.01 (FDR-adjusted *p* = 0.02), dominant *p* = 0.03 (FDR-adjusted *p* = 0.03), and recessive *p* = 0.01 (FDR-adjusted *p* = 0.02), and all remained statistically significant after FDR correction. In the codominant model (elite), mean LA30′ was highest for AA (10.77 mmol/L), intermediate for AG (9.54 mmol/L), and lowest for GG (6.96 mmol/L); in the dominant model, AA showed higher LA than (AG + GG); in the recessive model, (AA + AG) showed higher LA than GG. Analysis of rs1049434 demonstrated that the TT genotype had the highest LA concentrations in elite athletes (10.77 mmol/L), the TA carriers showed intermediate values (9.54 mmol/L), and the AA genotype had the lowest (6.96 mmol/L). Significant associations emerged under the codominant (*p* = 0.01; FDR-adjusted *p* = 0.02), dominant (*p* = 0.03; FDR-adjusted *p* = 0.03), and recessive (*p* = 0.01; FDR-adjusted *p* = 0.02) models, and all remained statistically significant after FDR correction. In the dominant model, TT showed higher LA compared with (TA + AA carriers), and in the recessive model, (TT + TA carriers) showed higher LA compared with AA (10.77 and 9.54 vs. 6.96 mmol/L). The rs10776763 polymorphism demonstrated significant associations in the elite group in all tested models. TT homozygotes showed the lowest LA concentrations (8.54 mmol/L), TC heterozygotes had intermediate values (10.48 mmol/L), and CC homozygotes the highest (13.02 mmol/L). The codominant model yielded *p* = 0.003 (FDR-adjusted *p* = 0.01), the dominant model *p* = 0.01 (FDR-adjusted *p* = 0.01), and the recessive model *p* = 0.002 (FDR-adjusted *p* = 0.01), and all remained statistically significant after FDR correction. Lastly, rs6537765 showed GG homozygotes in elite athletes exhibited the highest LA30′ concentrations (10.73 mmol/L), GA heterozygotes presented intermediate values (9.64 mmol/L), and AA homozygotes the lowest (6.96 mmol/L). Significant associations were observed across all genetic models in this group: codominant (*p* = 0.01; FDR-adjusted *p* = 0.02), dominant (*p* = 0.01; FDR-adjusted *p* = 0.02), and recessive (*p* = 0.04; FDR-adjusted *p* = 0.04), and all remained statistically significant after FDR correction (Table 5). Figure 2, Figure 3, Figure 4, Figure 5, Figure 6, Figure 7, Figure 8, Figure 9 and Figure 10 provide a time-course visualization of LA concentrations across genotypes and training groups, including LA30’.

## 4. Discussion

According to the SNP National Center for Biotechnology Information (NCBI) database [25], no publications are available regarding *MCT1* rs4301628, rs12028967, rs10857983, rs3789592, rs10776763, and rs6537765. The rs7556664 and rs7169 variants have been associated with improved survival outcomes in patients with multiple myeloma [26]. Nonetheless, rs1049434 has been linked to LA kinetics [12,17,20,27]. Our cross-sectional study, conducted in 2025 and involving 337 male athletes from Poland and the Czech Republic, appears to be the only published research to date that explicitly examines the association between nine selected *MCT1* polymorphisms and LA accumulation and removal in men performing high-intensity exercise [2]. Unlike the BMC Genomics study, which emphasized haplotype patterns and gene–gene interactions among MCT transporters, the current one uniquely investigates the independent effects of nine *MCT1* SNPs on specific post-exercise LA traits [2]. Employing SNP genotyping techniques, we analyzed the genetic factors associated with effective LA utilization among elite and sub-elite sprinters, as well as a control group of physically active males. We hypothesized that the selected *MCT1* gene variants would affect LA production and clearance in athletes whose training was characterized primarily by anaerobic effort.

In this research, the term LA accumulation denotes the increase in blood LA concentration, which typically occurs during exercise when the intensity exceeds the body’s clearance capacity [31]. Similarly to our earlier study [2], we evaluated blood LA concentrations in the present research. Some may argue that this methodology has limitations, noting that intramuscular LA is often regarded as yielding greater accuracy in representing metabolic state. The accumulation of LA primarily stems from increased pyruvate-to-LA conversion, which is modulated by shifts in the muscle’s redox balance. Moreover, the clearance of excess LA relies on its transport via the bloodstream to other working muscles, and to the heart and liver for further metabolism [32]. Therefore, LA concentrations within muscle tissue can serve as a valuable proxy for estimating systemic (blood) LA concentrations [33,34]. Importantly, elevated LA, whether measured in muscle or blood, reflects intensified proton production and a possible reduction in pH. Clinically, a rise in blood LA is widely recognized as a hallmark of tissue hypoxia [35]. To better interpret potential genotype–phenotype associations observed in this study, it is essential to consider the functional characteristics of the investigated variants. Seven of the investigated SNPs are located within intronic non-coding regions (rs4301628, rs12028967, rs10857983, rs3789592, rs7556664, rs10776763, rs6537765). Although introns can influence gene expression by altering splicing patterns or interfering with the binding of regulatory elements [36,37] it is unlikely that the investigated SNPs affect splicing, as no evidence supporting such effects is available in the Genotype-Tissue Expression Portal (GTEx) [38]. However, rs7169 is located in the non-coding 3′ untranslated region (3′UTR), and rs1049434 is a missense variant within the coding sequence [25]. Variants in the 3′UTR may affect mRNA stability, translational efficiency, and post-transcriptional regulation of gene expression [39]. In contrast, missense SNPs can directly alter the amino acid sequence of the encoded protein, potentially affecting its structure, function, and downstream cellular processes [40]. According to the GTEx portal, each of the intronic variants and rs7169 has expression quantitative trait loci (eQTL) data [38]. The non-coding variants modulate *MCT1* expression in the esophagus muscularis and spleen. Rs3789592, rs7556664, rs1049434, rs7169, and rs6537765 are also linked to *MCT1* expression in the heart [38]. Although expression effects are not reported for skeletal muscle, they provide indirect evidence that the variants may regulate *MCT1* expression in a tissue-specific manner. Given that LA is a systemic metabolite transported and utilized across multiple organs [41,42], these eQTL findings may support the potential regulatory role of the analyzed SNPs in LA metabolism beyond skeletal muscle. The available eQTL data for non-coding variants confirm changes in *MCT1* gene expression, which may affect the amount of protein produced and consequently influence LA transport. Unfortunately, no predictive data supporting the possible biological significance of non-coding variants to the *MCT1* function of these SNPs were identified. In contrast, the missense variant rs1049434 has been extensively studied and linked to LA metabolism and athletic performance, providing stronger evidence of biological relevance [11,17,18,19,20,22,27,28,29].

Upon evaluating rs4301628, rs12028967, rs10857983, rs3789592, rs7556664, rs7169, rs1049434, rs10776763, and rs6537765 polymorphisms, we identified significant associations between several of these polymorphisms and performance-related parameters.

For the *MCT1* rs4301628 polymorphism, a significant association with LA30’ was observed in the elite subgroup across all genetic models, including codominant (*p* = 0.004; FDR-adjusted *p* = 0.01), dominant, and recessive (both *p* = 0.01; FDR-adjusted *p* = 0.01) models. In elite, mean LA30′ values were CC = 8.73, CT = 10.35, TT = 13.02 mmol/L (TT highest). No significant associations were observed for LA30’ in the remaining subgroups (Table 5). As LA30’ reflects residual LA remaining in the bloodstream 30 min after exercise, this result may indicate less efficient LA clearance among TT homozygotes in elite athletes. In terms of MAX LA, no significant differences were found (Table 2). Regarding ACC, the association with the elite subgroup did not remain statistically significant following FDR correction (Table 3). For DCC, calculated as the difference between MAX LA and LA30’, no significant associations were found (Table 4). This may imply that rs4301628 modulates post-exercise LA30’ in elite athletes without a measurable effect on net LA removal during early recovery. This should be tested explicitly in larger, independent cohorts.

The *MCT1* rs12028967 variant showed a significant association with LA30’ in the elite subgroup across all genetic models: codominant (*p* = 0.004; FDR-adjusted *p* = 0.01), dominant and recessive (both *p* = 0.01; FDR-adjusted *p* = 0.01). In this group, GG homozygotes showed the highest LA30’ values (13.02 mmol/L), GT intermediate (10.35 mmol/L), and TT the lowest (8.73 mmol/L), which may suggest less efficient post-exercise LA clearance among elite athletes with the GG genotype. No significant associations were observed for LA30’ in the remaining subgroups (Table 5).

In terms of MAX LA, no significance was found (Table 2). For ACC, no associations survived FDR correction in any group (Table 3). For DCC, the variant was significant in the overall cohort (codominant *p* = 0.02; FDR-adjusted *p* = 0.03; recessive (TT + TG) vs. GG *p* = 0.02; FDR-adjusted *p* = 0.03), but not in the elite group (all *p* ≥ 0.08). In the entire cohort, TT + TG showed higher DCC than GG (8.14 and 8.07 vs. 7.26 mmol/L) (Table 4). Overall, the data are consistent with rs12028967 being associated with higher LA30′ in elite athletes across all genetic models, with no detectable effects on MAX LA or ACC and only a modest, cohort-level association with DCC. This pattern appears confined to the elite subgroup and warrants confirmation in larger samples.

*MCT1* rs10857983 polymorphism showed a significant association with LA30’ in the elite subgroup when analyzed across all genetic models: codominant (*p* = 0.004; FDR-adjusted *p* = 0.01), dominant and recessive (both *p* = 0.01; FDR-adjusted *p* = 0.01). In the codominant model (elite), mean LA30′ values were 8.73 mmol/L for CC, 10.35 mmol/L for CT, and 13.02 mmol/L for TT (Table 5). In this group, individuals with the TT genotype showed the highest LA30’ values, suggesting slower LA clearance in elite athletes with this genotype. No significant associations for LA30’ were noted in the other subgroups (Table 5). Regarding MAX LA, the TT genotype was also associated with the highest concentrations in the elite group, but no model reached significance after FDR (Table 2). For ACC, no associations survived FDR in any group (Table 3), i.e., nominal signals in the elite (dominant *p* = 0.03; FDR = 0.09) and in the total cohort (recessive *p* = 0.04; FDR = 0.12) did not persist. For DCC, no significant associations were observed for rs10857983 across any groups or models (Table 4), indicating limited evidence for its role in early post-exercise LA clearance. The rs10857983 pattern, with higher LA30′ in TT within the elite subgroup, suggests a possible modulation of early LA clearance. However, this remains speculative and warrants replication.

For the *MCT1* rs3789592 variant, a significant association with LA30’ was identified in the elite group across all genetic models: codominant (*p* = 0.01; FDR-adjusted *p* = 0.02), dominant (*p* = 0.03; FDR-adjusted *p* = 0.03), and recessive (*p* = 0.01; FDR-adjusted *p* = 0.02). GG showed the highest LA30′, AG was intermediate, and AA had the lowest (elite means: 10.77, 9.54, and 6.96 mmol/L, respectively) (Table 5). The highest LA30’ concentrations were observed among individuals with the GG genotype, while AA homozygotes showed the lowest values. These results may reflect genotype-related variation in residual LA concentrations 30 min after intense exercise in elite athletes (Table 5). For DCC, significant associations were also found in the elite group across codominant (*p* = 0.04; FDR-adjusted *p* = 0.04), dominant (*p* = 0.04; FDR-adjusted *p* = 0.04), and recessive (*p* = 0.04; FDR-adjusted *p* = 0.04) models. AA had the highest DCC (10.99 mmol/L), followed by AG (9.30 mmol/L) and GG (8.17 mmol/L) (Table 4). AA homozygotes had the highest DCC values, indicating a greater net reduction in LA during the recovery period. No FDR-significant associations for DCC were detected in the total sample or the physically active group (Table 4). For MAX LA and ACC, no significant associations were observed in any subgroup or genetic model after FDR correction (Table 2 and Table 3, respectively). This suggests that rs3789592 may not be a strong determinant of peak LA response or accumulation during high-intensity exercise. Its effects may be more relevant to LA regulation during the recovery phase, particularly in populations with a background in performance training.

For the rs7556664 polymorphism, significant differences in LA30’ were observed in the elite subgroup across all genetic models: codominant (*p* = 0.01; FDR-adjusted *p* = 0.02), dominant (*p* = 0.03; FDR-adjusted *p* = 0.03), and recessive (*p* = 0.01; FDR-adjusted *p* = 0.02). In elites, TT showed the lowest LA30′, AT intermediate, and AA the highest (means: 6.96, 9.54, 10.77 mmol/L) (Table 5). In addition, DCC was also significantly associated with rs7556664 in the elite subgroup across the same three genetic models: codominant, dominant, and recessive (*p* = 0.04 in all models; FDR-adjusted *p* = 0.04 for each model), with TT showing the highest DCC (10.99 mmol/L), AT 9.30 mmol/L, and AA 8.17 mmol/L. In the sub-elite subgroup, the recessive model was initially significant (*p* = 0.03) but did not survive FDR (FDR-adjusted *p* = 0.09). No significant associations were identified for DCC in the total sample or among physically active individuals (Table 4). No associations between rs7556664 and MAX LA (Table 2) or ACC (Table 3) survived FDR in any subgroup or model. Collectively, the rs7556664 pattern in elite athletes, i.e., lower LA30 alongside higher DCC in TT, aligns with more efficient early LA clearance rather than altered peak production. Again, effects should be tested explicitly in larger, independent cohorts with prespecified interaction analyses.

For the *MCT1* rs7169 polymorphism, a significant association with LA30’ was identified in the elite subgroup across all tested genetic models: codominant (*p* = 0.01; FDR-adjusted *p* = 0.02), dominant (*p* = 0.03; FDR-adjusted *p* = 0.03), and recessive (*p* = 0.01; FDR-adjusted *p* = 0.02). In this group, the GG genotype showed the lowest LA30′, AG was intermediate, and AA had the highest values (elite codominant: AA = 10.77, AG = 9.54, GG = 6.96 mmol/L). No significant LA30′ associations were detected in other subgroups (Table 5). Consistent with a clearance-phase signal, DCC in elites was also significant across all three models (*p* = 0.04; FDR-adjusted *p* = 0.04), with AA and AG lower than GG (elite: AA = 8.17, AG = 9.30, GG = 10.99 mmol/L). In the sub-elite, the recessive model was nominally significant (*p* = 0.03), but it did not survive FDR (FDR-adjusted *p* = 0.09). No DCC associations were significant in the total or physically active groups (Table 4). No associations for MAX LA (Table 2) or ACC (Table 3) survived FDR in any subgroup for rs7169. Taken together, rs7169 showed FDR-significant associations with lower residual LA (LA30′) and higher net reduction (DCC) in the elite subgroup; this pattern is consistent with more efficient early post-exercise clearance in the GG genotype compared with the AA/AG genotype. Given that rs7169 is positioned in the 3′UTR of the *MCT1* gene, one possible mechanism could involve altered post-transcriptional regulation of MCT1 expression, which may affect the efficiency of LA transport during recovery. Further mechanistic studies would be valuable to determine whether rs7169 affects mRNA stability or translation of *MCT1* in a way that influences LA handling following intense anaerobic exertion.

For the *MCT1* rs1049434 variant, a significant association with LA30’ was observed in the elite subgroup across all genetic models: codominant (*p* = 0.01; FDR-adjusted *p* = 0.02), dominant (*p* = 0.03; FDR-adjusted *p* = 0.03), and recessive (*p* = 0.01; FDR-adjusted *p* = 0.02). In elites, AA showed the lowest LA30′ (6.96 mmol/L), AT intermediate (9.54 mmol/L), and TT the highest (10.77 mmol/L). No LA30′ associations were significant in other subgroups. (Table 5). DCC was also significant in elites across codominant, dominant, and recessive models (*p* = 0.04; FDR-adjusted *p* = 0.04 for each), with AA showing the highest DCC (10.99 mmol/L), AT 9.30 mmol/L, and TT 8.17 mmol/L. In the sub-elite, the recessive model was nominally significant (*p* = 0.03) but did not survive FDR (FDR-adjusted *p* = 0.09). No DCC associations were significant in the total or physically active groups (Table 4). No rs1049434 associations with MAX LA (Table 2) or ACC (Table 3) survived FDR in any subgroup. This pattern, i.e., lower LA30′ and higher DCC for AA in elites, appears consistent with more efficient early post-exercise LA clearance linked to rs1049434 in this subgroup. Given that rs1049434 is a missense variant, a functional effect on transporter behavior is plausible but remains speculative. Replication in larger, independent elite cohorts along with functional follow-up, is warranted.

For *MCT1* rs10776763 polymorphism, a significant association with LA30’ was observed in the elite subgroup across all genetic models: codominant (*p* = 0.003; FDR-adjusted *p* = 0.01), dominant (*p* = 0.01; FDR-adjusted *p* = 0.01), and recessive (*p* = 0.002; FDR-adjusted *p* = 0.01). In this subgroup, CC had the highest LA30′ (13.02 mmol/L), TC intermediate (10.48 mmol/L), and TT the lowest (8.54 mmol/L). No LA30′ associations were significant in other subgroups (Table 5). These findings may indicate genotype-related variation in LA removal efficiency, with the CC genotype potentially linked to slower post-exercise clearance in elite athletes. DCC showed nominal associations for rs10776763 in elites (codominant *p* = 0.03; FDR-adjusted *p* = 0.09) and in the total cohort under the recessive model (*p* = 0.03; FDR-adjusted *p* = 0.09). Neither survived FDR adjustment (Table 4). ACC was significant in elites under the recessive model (*p* = 0.01; FDR-adjusted *p* = 0.03): TT + TC showed lower ACC than CC (15.82 and 17.08 vs. 17.67 mmol/L). No ACC associations reached FDR significance in other groups (Table 3). Regarding DCC, no associations with rs10776763 survived FDR in any subgroup (Table 4). These findings suggest elite-specific differences in LA maintenance linked to rs10776763, but confirmation in larger, independent elite cohorts is warranted. Further studies are necessary to confirm the functional consequences of this variant.

For the *MCT1* rs6537765 variant, a significant association with LA30’ was identified in the elite subgroup across all genetic models: codominant, dominant (both *p* = 0.01 and both FDR-adjusted *p* = 0.02), and recessive (*p* = 0.04; FDR-adjusted *p* = 0.04). In elites, GG showed the highest LA30′, AG intermediate, and AA the lowest (means: AA = 6.96, AG = 9.64, GG = 10.73 mmol/L). No LA30′ associations were significant in other subgroups (Table 5). Specifically, the AA genotype may be linked to more efficient post-exercise LA removal in elite athletes. DCC was also significant in elites across codominant, dominant, and recessive models (*p* = 0.04; FDR-adjusted *p* = 0.04 for each), with AA showing the highest DCC, suggesting a greater net reduction in LA from peak to 30 min post-exercise compared to other genotypes, AG intermediate, and GG the lowest (means: AA = 10.99, AG = 9.28, GG = 8.14 mmol/L). In the sub-elite, the recessive model showed *p* = 0.03; FDR-adjusted *p* = 0.45 (not significant after FDR), and no DCC associations reached significance in the total or physically active groups (overall recessive *p* = 0.03; FDR-adjusted *p* = 0.09) (Table 4). No significant associations were found between rs6537765 and MAX LA (Table 2) or ACC (Table 3) in any subgroup or genetic model. This suggests that rs6537765 is relevant for early post-exercise LA clearance rather than peak production. It may be involved in interindividual differences in post-exercise LA handling, especially in athletes with a high training background. Additional studies, particularly functional ones, are needed to determine whether this variant influences the expression or activity of MCT1 and how it might contribute to recovery efficiency or performance outcomes.

In this current study, we focused on MAX LA, ACC, DCC, and final LA30’. In our previous work, we emphasized that ACC measures the degree of increase in LA concentration during physical activity. A higher ACC suggests a superior capacity to generate or tolerate LA, which can be beneficial during brief, high-intensity efforts that primarily rely on anaerobic metabolism. Conversely, excessive LA accumulation may indicate a reduced ability to clear or transport LA efficiently. DCC, on the other hand, denotes the organism’s ability to utilize and eliminate LA from the blood in the post-exercise period [30]. When we restricted inference to FDR-corrected results, the most consistent signals in our dataset clustered in the clearance phase and, critically, within the elite subgroup. Five variants—rs3789592, rs7556664, rs7169, rs1049434, and rs6537765—each showed FDR-significant effects on both LA30′ and DCC in elites, indicating reproducible associations across two complementary removal metrics in the same group. In addition, rs10776763 was the only polymorphism that spanned phases in elites, with LA30′ surviving FDR in all models and ACC surviving FDR in the recessive model, making it the broadest multi-aspect signal. Rs12028967 also supported a clearance theme but was split across cohorts (LA30′ FDR-significant in elites, DCC FDR-significant in the total cohort). By contrast, rs4301628 and rs10857983 demonstrated single-phenotype clearance effects (LA30′) confined to elites, and no production-phase endpoint (MAX LA or ACC) other than rs10776763 survived FDR. These patterns might explain why several associations appeared only in the smallest (elite) subgroup. Training status likely concentrates physiologic homogeneity and effect expression, while heterogeneity in the larger groups dilutes detectable effects. Nevertheless, one signal (rs12028967 for DCC) was discernible at the cohort level. Collectively, the FDR-adjusted evidence suggests that *MCT1* variation in this study is preferentially related to post-exercise LA clearance, most consistently in elite athletes, rather than to peak production. This finding motivates prospective tests of the genotype × training-status interaction in larger, independent samples. Notably, in our previous work, genotype effects were group-specific—some confined to elites, some detectable only in the total cohort, and others limited to physically active individuals [30]. In the present dataset, after FDR correction, signals converged predominantly in elites and in the clearance phase (LA30′ and DCC), with one cohort-level clearance effect (rs12028967 on DCC) also detectable. The pattern in elites is compatible with effect modification by training status, as exercise induces molecular changes [43]. Therefore, the revealed genotype influences on recovery-phase LA kinetics in elite athletes cannot be excluded. In contrast, greater heterogeneity in broader groups can attenuate marginal effects [44], but this is not always the case [30]. Simultaneously, we acknowledge that sample size is a limitation in the elite subgroup (discussed in the Limitations section) and that subgroup-restricted findings must be approached cautiously. At the same time, several features argue against a purely chance, subgroup-specific artifact: (i) five variants show convergent, FDR-significant associations on two clearance endpoints (LA30′ and DCC) within elites, (ii) directions of effect are internally consistent across models, (iii) one clearance signal (rs12028967 for DCC) is detectable at the cohort level, and (iv) biologically, training status is a plausible effect modifier for *MCT1*-mediated LA transport [45]. Nonetheless, residual sampling error cannot be entirely excluded. Therefore, we recommend the need for larger, independent cohorts with pre-specified interaction testing, alongside sensitivity analyses. The observed genotype-dependent variation in post-exercise LA handling may reflect functional differences in *MCT1* transport efficiency. The MCT1 transporter exhibits relatively modest affinity toward LA (Michaelis constant, Km 3–5 mM) and operates more effectively when LA concentrations are elevated [14]. In this context, polymorphism such as *rs10776763*, which was significantly associated with LA30′ (across models in elites) and with ACC in elites under the recessive model, might influence *MCT1* expression, localization, or transport kinetics, either directly or through linkage with a functional variant. This could help explain the differences in LA accumulation and clearance observed between genotypes. While the functional consequences of this SNP remain to be established, the current findings raise the possibility that interindividual variation in LA dynamics may, in part, reflect differences in how effectively *MCT1* responds under high-LA conditions.

The present findings partially align with our previous results reported in our BMC Genomics study, which focused on gene–gene interactions across MCT family members [2]. In that analysis, several *MCT1* SNPs investigated here, namely rs3789592, rs12028967, rs7556664, rs10857983, and rs4301628, emerged as components of significant genotype combinations associated with post-exercise LA outcomes, such as the difference between maximum and final LA concentration (DCC). While the previous analysis did not assess the independent effects of these variants, the present study extends those observations by demonstrating that several of the same loci show FDR-significant main effects—predominantly on clearance-phase phenotypes (LA30′ and/or DCC) and mainly within the elite subgroup—and, for rs12028967, a cohort-level DCC signal. For example, rs3789592 and rs12028967 were previously implicated in gene–gene interactions linked to elevated LA differences [30]. In the present dataset, both map to the clearance phase: rs3789592 showed FDR-significant associations with LA30′ and DCC in elites, whereas rs12028967 showed FDR-significant associations with LA30′ in elites and FDR-significant associations with DCC in the overall cohort. Neither variant showed FDR-significant signals for MAX LA or ACC. Likewise, rs4301628 and rs10857983, which were previously linked to lower MAX LA within specific gene-gene combinations [30], exhibited FDR-significant LA30′ effects confined to elites in the current study, with no FDR-significant production-phase (MAX LA or ACC) or DCC effects. Taken together, these complementary findings suggest that specific *MCT1* polymorphisms may influence post-exercise LA clearance kinetics not only via interactions with other MCT variants, but also through their individual effects. Future work integrating single-variant and interaction models, alongside functional assays, will be important to define their physiological impact.

### Limitations

The limited availability of literature creates challenges in finding relevant data. However, our findings add to the current understanding, as our work explores new areas. On the contrary, this research was unable to offer insights concerning Polish and Czech females, and this remains an important subject for future research. There is overwhelming evidence that women are underrepresented in the field of sports science [46,47,48,49]. The decision to focus exclusively on men in this study was influenced by the scope of the intervention and the small number of women who were accessible at that time. Moreover, recruiting female athletes for a demanding protocol involving two consecutive Wingate tests posed practical challenges, which limited the feasibility of including them in the present analysis. Future replication studies should extend to larger and independent athlete cohorts, including female participants, to determine whether the observed associations are consistent across sexes. A notable constraint of this study is the small participant pool, comprising only 42 elite athletes. Recruiting elite athletes presents considerable logistical and practical challenges due to their limited availability, rigorous training commitments, and selective recruitment processes. At the time of recruitment, this number essentially reflected the whole pool of accessible Polish and Czech elite sprinters, making substantial enlargement of this group unfeasible. The very low counts in some genotype categories (3–4 individuals) are a direct consequence of the combination of allele frequencies and the limited size of the elite cohort. The 42 elite sprinters form a distinct and carefully selected group, which limits attempts to enhance the number of participants substantially. Another limitation is that we did not formally test for population stratification between Polish and Czech participants. However, all individuals reported Caucasian/European ancestry, as self-reported, from neighboring Central European countries. Previous genome-wide SNP studies have demonstrated that Poles and Czechs cluster within the broader “northern/central European” genetic group, showing only minimal allele frequency differences compared to each other, especially when contrasted with the much stronger north–south differentiation observed within Europe [50]. Therefore, the likelihood that undetected population substructure between Polish and Czech participants confounded our results is expected to be low. Research conducted in more ethnically diverse populations is needed to assess whether these factors contribute to variation in the results. Moreover, conducting a replication study with a larger sample size is imperative to assess the reliability and consistency of the observed effects in our study. To validate the observed associations and increase generalizability, replication in larger and more ethnically diverse athlete populations is essential. Future studies should also investigate genotype × training level interactions in larger cohorts to clarify whether the observed subgroup-specific associations reflect true biological interactions or are due to limitations in sample size. The cross-sectional design can demonstrate associations but is unable to establish a cause-and-effect relationship between genetic variations and LA kinetics. Cultural factors such as diet, physical activity habits, and training methods may influence the expression or functionality of MCT transporter variants. For example, culturally specific dietary habits could either support or interfere with effective LA transport, thereby influencing both LA accumulation and clearance. A more detailed investigation of these interactions is warranted in future research to better understand their role in shaping individual LA profiles. Next, this study is based exclusively on genotype–phenotype associations and does not include direct molecular assessments, such as measurements of gene expression or transporter function at the protein level. While this limits the ability to draw mechanistic conclusions, we attempted to mitigate this gap by analyzing physiological indicators of LA metabolism (LA30’, MAX LA, ACC, and DCC) obtained from repeated Wingate tests. Although these markers do not replace molecular validation, they provide functional insights into the potential physiological impact of genetic variation. To enhance mechanistic understanding, future research should incorporate molecular techniques or gene expression analysis to evaluate MCT1 function more directly in relation to genotype.

The findings reported here are based on a maximal anaerobic effort. The Wingate test, a well-established protocol, was selected to elicit high levels of LA production and to assess anaerobic performance under standardized conditions. While effective, this test requires correct pedaling technique on a cycle ergometer, which may be seen as a limitation. In individuals with limited cycling experience or coordination challenges, performance outcomes could reflect motor skill as much as anaerobic capacity. Despite this, we believe the Wingate test was an appropriate choice to evaluate LA utilization and anaerobic output. Notably, prior studies have demonstrated that just one Wingate sprint can reduce muscle glycogen content in the vastus lateralis by approximately 20–30% [51,52,53,54,55], highlighting the firm reliance on anaerobic metabolism during this task.

## 5. Conclusions

The present study indicated that genetic variability in the *MCT1* gene is more consistently associated with post-exercise LA removal than with peak lactate production. After FDR correction, associations were detected in the elite subgroup, where five variants (rs3789592, rs7556664, rs7169, rs1049434, and rs6537765) remained significant for both LA30′ and DCC. One variant, rs10776763, extended across phases, remaining significant for LA30′ in all models and for ACC in the recessive model, suggesting a broader role in both LA buildup and removal. Rs12028967 also supported a clearance-related role, with effects distributed across cohorts. In contrast, rs4301628 and rs10857983 demonstrated isolated associations confined to LA30′ in elites, and no production-phase endpoint other than rs10776763 survived FDR adjustment. Together, these findings suggest that *MCT1* variation influences the recovery phase of LA kinetics in athletes, particularly those with advanced training status, more than it affects maximal LA production. This pattern highlights the potential modifying role of training background on how genetic variation in *MCT1* translates into LA kinetics. Future studies should replicate these subgroup-specific signals in larger and more diverse athlete cohorts, integrate functional assays to clarify underlying mechanisms, and explicitly test genotype × training interactions. Such work will be essential for determining whether *MCT1* variants can inform individualized recovery strategies or talent identification in high-intensity sports.

## Figures and Tables

**Table 1:** Genotype frequencies in *MCT1* gene polymorphisms and HWE *p*-values.

MCT1 SNP				AA (%)				Aa (%)				aa (%)				HWE p-Values			
	A	a	MAF (%)	All	Elite	Sub-Elite	Physically Active	All	Elite	Sub-Elite	Physically Active	All	Elite	Sub-Elite	Physically Active	All	Elite	Sub-Elite	Physically Active
rs4301628	C	T	33.23	43.26	50.00	43.69	43.36	45.39	42.86	49.51	38.94	11.35	7.14	6.80	17.70	0.90	1.00	0.18	0.07
rs12028967	T	G	33.09	42.91	50.00	43.69	42.48	45.74	42.86	49.51	39.82	11.35	7.14	6.80	17.70	0.89	1.00	0.18	0.11
rs10857983	C	T	33.23	43.26	50.00	43.69	43.36	45.39	42.86	49.51	38.94	11.34	7.14	6.80	17.70	0.90	1.00	0.18	0.07
rs3789592	G	A	37.39	39.36	45.24	33.98	43.36	46.45	42.86	52.43	39.82	14.18	11.90	13.59	16.81	0.90	1.00	0.41	0.16
rs7556664	A	T	37.54	39.36	45.24	33.98	43.36	46.45	42.86	52.43	39.82	14.18	11.90	13.59	16.81	0.90	1.00	0.41	0.16
rs7169	A	G	37.54	39.01	45.24	33.98	42.48	47.16	42.86	52.43	41.59	13.83	11.90	13.59	15.92	1.00	1.00	0.41	0.31
rs1049434	T	A	37.54	39.36	45.24	33.98	43.36	46.45	42.86	52.43	39.82	14.18	11.90	13.59	16.81	0.90	1.00	0.41	0.16
rs10776763	T	C	35.46	40.78	47.62	38.83	42.48	47.16	45.24	53.4	38.05	12.06	7.14	7.77	19.47	0.70	0.73	0.08	0.05
rs6537765	G	A	37.69	38.30	42.86	33.01	42.48	47.52	45.24	53.4	39.82	14.18	11.90	13.59	17.70	1.00	1.00	0.31	0.11

A and a are symbolic representations of alleles: A—major, a—minor, AA = homozygous major, Aa = heterozygous, aa = homozygous minor.

**Table 2:** Blood LA mean maximum values (MAX LA) in mmol/L for all, physically active, elite, and sub-elite individuals across genotypes in *MCT1* polymorphisms, analyzed under codominant, dominant, and recessive allele models, with their effects (η^2^, d) with 95% CI and post hoc power.

*MCT1* SNP	Genotype	All	Physically Active	Sub-Elite	Elite	Model	All *p*-Value	Physically Active *p*-Value	Sub-Elite *p*-Value	Elite *p*-Value	All Effect (η^2^, d); (95% CI)	Physically Active Effect (η^2^, d); (95% CI)	Sub-Elite Effect (η^2^, d); (95% CI)	Elite Effect (η^2^, d); (95% CI); Post Hoc Power
rs4301628	CT	16.40	15.09	17.46	19.14	codominant	0.07	0.62	0.82	0.16	0.002; (0.00 0.03)	0.01; (0.00 0.03)	0.01; (0.00 0.07)	0.09; (0.00 0.27)
	CC	16.53	15.43	17.82	18.24	dominant	0.07	0.36	0.54	0.08	0.07; (−0.15 0.28)	0.13; (−0.15 0.42)	0.16; (−0.22 0.56)	−0.61; (−1.23 0.01)
	TT	16.14	15.26	17.64	20.05	recessive	0.48	1.00	0.99	0.19	0.12; (−0.22 0.46)	<0.001; (−0.40 0.40)	−0.17; (−0.93 0.60)	−0.53; (−1.71 0.65)
rs12028967	GT	16.40	15.08	17.46	19.14	codominant	0.28	0.59	0.82	0.16	0.002; (0.00 0.03)	0.01; (0.00 0.04)	0.01; (0.00 0.07)	0.09; (0.00 0.27)
	TT	16.53	15.43	17.82	18.24	dominant	0.75	0.34	0.54	0.08	−0.07; (−0.28 0.15)	−0.14; (−0.42 0.15)	−0.17; (−0.56 0.22)	0.61; (−0.01 1.23)
	GG	16.14	15.26	17.64	20.05	recessive	0.25	1.00	0.99	0.19	−0.12; (−0.46 0.22)	<0.001; (−0.40 0.40)	0.17; (−0.60 0.93)	0.53; (−0.65 1.71)
rs10857983	CT	16.40	15.09	17.46	19.14	codominant	0.04/0.06 *	0.62	0.82	0.16	0.002; (0.00 0.03)	0.01; (0.00 0.03)	0.01; (0.00 0.07)	0.09; (0.00 0.27)
	CC	16.53	15.43	17.82	18.24	dominant	0.60	0.36	0.54	0.08	0.07; (−0.15 0.28)	0.13; (−0.15 0.42)	0.17; (−0.22 0.56)	−0.61; (−1.23 0.01)
	TT	16.14	15.26	17.64	20.05	recessive	0.03/0.06 *	1.00	0.99	0.19	0.12; (−0.22 0.45)	<0.001; (−0.40 0.40)	−0.17; (−0.93 0.60)	−0.53; (−1.71 0.65)
rs3789592	AG	16.74	15.48	18.01	18.84	codominant	0.05	0.42	0.14	0.62	0.02 (0.00 0.10)	0.01; (0.00 0.05)	0.02; (0.00 0.10)	0.07; (0.00 0.24)
	GG	16.34	15.17	17.54	18.94	dominant	0.61	0.63	0.81	0.64	0.05; (−0.17 0.27)	0.07; (−0.22 0.36)	−0.04; (−0.45 0.37)	0.003; (−0.61 0.61)
	AA	15.64	14.89	16.39	17.95	recessive	0.03/0.08 *	0.32	0.07	0.33	−0.34; (−0.64 −0.03)	−0.20; (−0.60 0.20)	−0.44; (−1.01 0.13)	−0.78; (−1.72 0.18)
rs7556664	AT	16.75	15.51	18.01	18.84	codominant	0.04/0.06 *	0.34	0.14	0.62	0.02 (0.00 0.10)	0.01; (0.00 0.05)	0.02; (0.00 0.10)	0.07; (0.00 0.24)
	TT	15.64	14.89	16.39	17.95	dominant	0.60	0.50	0.81	0.64	−0.06; (−0.28 0.16)	−0.10; (−0.39 0.19)	0.04; (−0.37 0.45)	−0.003; (−0.61 0.61)
	AA	16.33	15.13	17.54	18.94	recessive	0.03/0.06 *	0.32	0.07	0.33	0.34; (0.03 0.64)	0.20; (−0.20 0.60)	0.44; (−0.13 1.01)	0.78; (−0.18 1.72)
rs7169	AG	16.74	15.52	18.01	18.84	codominant	0.05	0.31	0.14	0.62	0.02 (0.00 0.10)	0.01; (0.00 0.05)	0.02; (0.00 0.10)	0.07; (0.00 0.24)
	GG	13.40	14.86	16.39	17.95	dominant	0.65	0.47	0.81	0.64	−0.06; (−0.28 0.16)	−0.11; (−0.40 0.18)	0.04; (−0.37 0.45)	−0.003; (−0.61 0.61)
	AA	16.33	15.12	17.54	18.94	recessive	0.03/0.08 *	0.31	0.07	0.33	0.33; (0.02 0.64)	0.21; (−0.19 0.61)	0.44; (−0.13 1.01)	0.78; (−0.18 1.72)
rs1049434	AT	16.75	15.51	18.01	18.84	codominant	0.73	0.34	0.14	0.62	0.02 (0.00 0.10)	0.01; (0.00 0.05)	0.02; (0.00 0.10)	0.07; (0.00 0.24)
	TT	16.33	15.13	17.54	18.94	dominant	0.54	0.50	0.81	0.64	0.06; (−0.16 0.28)	0.10; (−0.19 0.39)	−0.04; (−0.45 0.37)	0.003; (−0.61 0.61)
	AA	15.64	14.89	16.39	17.95	recessive	0.50	0.32	0.07	0.33	−0.34 (−0.64 0.03)	−0.20; (−0.60 0.20)	−0.44; (−1.01 0.13)	−0.78; (−1.72 0.18)
rs10776763	TC	16.50	15.21	17.50	19.24	codominant	0.73	0.52	0.71	0.08	0.004; (0.00 0.05)	0.002; (0.00 0.02)	0.01; (0.00 0.07)	0.13; (0.00 0.32)
	CC	15.97	15.10	17.39	20.05	dominant	0.55	0.66	0.81	0.19	−0.03; (−0.25 0.18)	−0.09; (−0.38 0.19)	−0.19; (−0.59 0.21)	0.75; (0.12 1.38)
	TT	16.48	15.39	17.85	18.10	recessive	0.50	0.52	0.52	0.03/0.09 *	−0.19; (−0.52 0.14)	−0.09; (−0.48 0.30)	0.05; (−0.67 0.77)	0.53; (−0.65 1.71); 0.75
rs6537765	AG	16.74	15.54	17.85	18.92	codominant	0.73	0.26	0.40	0.63	0.02; (0.00 0.09)	0.01; (0.00 0.06)	0.01; (0.00 0.07)	0.08; (0.00 0.25)
	GG	16.30	15.10	17.64	18.87	dominant	0.54	0.31	0.19	0.33	0.08; (−0.14 0.30)	0.13; (−0.16 0.41)	−0.10; (−0.52 0.31)	0.09; (−0.53 0.70)
	AA	15.75	14.86	16.74	17.95	recessive	0.50	0.39	0.97	0.81	−0.29; (−0.60 0.02)	−0.21; (−0.61 0.19)	−0.29; (−0.86 0.28)	−0.78; (−1.72 0.18)

*MCT1*—monocarboxylate transporter 1, * false discovery rate (FDR) correction; CI—confidence interval.

**Table 5:** Blood LA mean final LA values in mmol/L measured at 30 min after the second Wingate (LA30’) for all, physically active, elite, and sub-elite, across genotypes in *MCT1* polymorphisms, analyzed under the codominant, dominant, and recessive allele models, with their effects (η^2^, d) with 95% CI and post hoc power.

*MCT1* SNP	Genotype	All	Physically Active	Sub-Elite	Elite	Model	All *p*-Value	Physically Active *p*-Value	Sub-Elite *p*-Value	Elite *p*-Value	All Effect (η^2^, d); (95% CI)	Physically Active Effect (η^2^, d); (95% CI)	Sub-Elite Effect (η^2^, d); (95% CI)	Elite Effect (η^2^, d); (95% CI); Post Hoc Power
rs4301628	CT	8.25	7.44	8.77	10.35	codominant	0.40	0.37	0.42	0.004/0.01 *	0.005; (0.00 0.05)	0.01; (0.00 0.05)	0.02; (0.00 0.09)	0.16; (0.00 0.36); 0.95
	CC	8.46	7.80	9.60	8.73	dominant	0.21	0.69	0.21	0.01/0.01 *	0.03; (−0.19 0.24)	0.06; (−0.23 0.34)	0.19; (−0.20 0.59)	−0.71; (−1.32 −0.08); 0.80
	TT	8.88	8.22	9.23	13.02	recessive	0.92	0.26	0.95	0.01/0.01 *	−0.18; (−0.52 0.16)	−0.23; (−0.63 0.17)	−0.27; (−1.04 0.50)	−1.21; (−2.4 −0.002); 0.82
rs12028967	GT	8.26	7.44	8.77	10.35	codominant	0.40	0.36	0.42	0.004/0.01 *	0.004; (0.00 0.05)	0.01; (0.00 0.05)	0.02; (0.00 0.09)	0.16; (0.00 0.36); 0.95
	TT	8.46	7.80	9.60	8.73	dominant	0.66	0.68	0.21	0.01/0.01 *	−0.03; (−0.24 0.19)	−0.06; (−0.34 0.23)	−0.19; (−0.59 0.20)	0.71; (0.08 1.33); 0.80
	GG	8.88	8.22	9.23	13.02	recessive	0.42	0.26	0.95	0.01/0.01 *	0.18; (−0.16 0.52)	<0.001; (−0.40 0.40)	0.27; (−0.50 1.04)	1.21; (0.00 2.41); 0.82
rs10857983	CT	8.25	7.44	8.77	10.35	codominant	0.34	0.37	0.42	0.004/0.01 *	0.005; (0.00 0.05)	0.01; (0.00 0.05)	0.02; (0.00 0.09)	0.16; (0.00 0.36); 0.95
	CC	8.46	7.80	9.60	8.73	dominant	0.87	0.69	0.21	0.01/0.01 *	0.03; (−0.19 0.24)	0.06; (−0.23 0.34)	0.20; (−0.20 0.59)	−0.71; (−1.32 −0.08); 0.80
	TT	8.88	8.22	9.23	13.02	recessive	0.18	0.26	0.95	0.01/0.01 *	−0.18; (−0.52 0.16)	−0.23; (−0.63 0.17)	−0.27; (−1.04 0.50)	−1.21; (−2.41 −0.002); 0.82
rs3789592	AG	8.58	8.01	9.16	9.54	codominant	0.36	0.38	0.99	0.01/0.02 *	0.006; (0.00 0.06)	0.01; (0.00 0.05)	0.01; (0.00 0.52)	0.09; (0.00 0.27); 0.90
	GG	8.41	7.48	9.20	10.77	dominant	0.84	0.29	0.94	0.03/0.03 *	0.004; (−0.22 0.22)	0.15; (−0.13 0.44)	−0.14; (−0.55 0.27)	−0.43; (−1.04 0.19); 0.54
	AA	7.91	7.50	9.09	6.96	recessive	0.20	0.63	0.93	0.01/0.02 *	−0.21; (−0.51 0.10)	−0.10; (−0.49 0.30)	−0.16; (−0.73 0.40)	−0.83; (−1.78 0.12); 0.54
rs7556664	AT	8.60	8.05	9.16	9.54	codominant	0.34	0.26	0.99	0.01/0.02 *	0.007; (0.00 0.06)	0.01; (0.00 0.06)	0.01; (0.00 0.52)	0.09; (0.00 0.27); 0.90
	TT	7.91	7.50	9.09	6.96	dominant	0.87	0.20	0.94	0.03/0.03 *	−0.02; (−0.24 0.20)	−0.19; (−0.48 0.10)	0.14; (−0.27 0.55)	0.43; (−0.19 1.04); 0.54
	AA	8.39	7.42	9.20	10.77	recessive	0.18	0.63	0.93	0.01/0.02 *	0.21; (−0.10 0.51)	0.10; (−0.30 0.49)	0.16; (−0.40 0.73)	0.83; (−0.12 1.78); 0.54
rs7169	AG	8.60	8.06	9.16	9.54	codominant	0.36	0.23	0.99	0.01/0.02 *	0.006; (0.00 0.06)	0.02; (0.00 0.06)	0.01; (0.00 0.05)	0.09; (0.00 0.27); 0.90
	GG	7.92	7.51	9.09	6.96	dominant	0.97	0.17	0.94	0.03/0.03 *	−0.02; (−0.24 0.20)	−0.20; (−0.49 0.08)	0.14; (−0.27 0.55)	0.43; (−0.19 1.04); 0.54
	AA	8.38	7.40	9.20	10.77	recessive	0.18	0.65	0.93	0.01/0.02 *	0.20; (−0.11 0.51)	0.21; (−0.19 0.61)	0.16; (−0.40 0.73)	0.83; (−0.12 1.78); 0.54
rs1049434	AT	8.60	8.05	9.16	9.54	codominant	0.47	0.26	0.99	0.01/0.02 *	0.007; (0.00 0.06)	0.01; (0.00 0.06)	0.01; (0.00 0.05)	0.09; (0.00 0.27); 0.90
	TT	8.39	7.42	9.20	10.77	dominant	0.80	0.20	0.94	0.03/0.03 *	0.02; (−0.20 0.24)	0.19; (−0.10 0.48)	−0.14; (−0.55 0.27)	−0.43; (−1.04 0.19); 0.54
	AA	7.91	7.50	9.09	6.96	recessive	0.29	0.63	0.93	0.01/0.02 *	−0.21; (−0.51 0.10)	−0.10; (−0.49 0.30)	−0.16; (−0.73 0.40)	−0.83; (−1.78 0.12); 0.54
rs10776763	TC	8.40	7.53	9.00	10.48	codominant	0.47	0.42	0.66	0.003/0.01 *	0.002; (0.00 0.03)	0.01; (0.00 0.04)	0.004; (0.00 0.04)	0.20; (0.01 0.40); 0.99
	CC	8.76	8.17	8.85	13.02	dominant	0.81	0.29	0.79	0.01/0.01 *	0.05; (−0.17 0.27)	−0.01; (−0.30 0.28)	−0.09; (−0.49 0.31)	0.85; (0.21 1.48); 0.91
	TT	8.34	7.73	9.43	8.54	recessive	0.29	0.93	0.49	0.002/0.01 *	0.14; (−0.19 0.46)	0.21; (−0.18 0.60)	0.12; (−0.60 0.85)	1.21; (0.002 2.41); 0.82
rs6537765	AG	8.58	8.06	9.02	9.64	codominant	0.47	0.24	0.84	0.01/0.02 *	0.006; (0.00 0.06)	0.02; (0.00 0.06)	0.01; (0.00 0.08)	0.08; (0.00 0.26); 0.89
	GG	8.40	7.40	9.41	10.73	dominant	0.80	0.65	0.96	0.01/0.02 *	0.01; (−0.21 0.23)	0.20; (−0.09 0.49)	−0.25; (−0.66 −0.17)	−0.36; (−0.97 0.26); 0.40
	AA	7.93	7.51	9.13	6.96	recessive	0.29	0.17	0.57	0.04/0.04 *	−0.20; (−0.51 0.11)	−0.09; (−0.50 0.31)	−0.15; (−0.71 0.41)	−0.83; (−1.78 0.12); 0.54

*MCT1*—monocarboxylate transporter 1, * false discovery rate (FDR) correction; CI—confidence interval.

## Data Availability

The original data presented in this study are openly available in the European Variation Archive (EVA) at EMBL-EBI under the accession number PRJEB85239 (https://www.ebi.ac.uk/eva/?eva-study=PRJEB85239) (accessed on 28 January 2025).

## Supplementary Materials

The following supporting information can be downloaded at: https://www.mdpi.com/article/10.3390/genes16101160/s1, Supplementary Table S1. LArest—LA at rest, LAaftwarm—LA following the warm-up, LABETWEEN-LA during the third minute of rest after the first Wingate test but before the second, LAaft2Want—LA immediately after the second Wingate; LA3’—LA at 3’ min post-second Wingate; LA6’—LA at 6’ min post-second Wingate, LA9’—LA at 9’ min post-second Wingate, LA20’—LA at 20’ min post-second Wingate; LA30’—LA at 30’ min after the second Wingate test.
